# An Ultrasound Image-Based Dynamic Fusion Modeling Method for Predicting the Quantitative Impact of *In Vivo* Liver Motion on Intraoperative HIFU Therapies: Investigations in a Porcine Model

**DOI:** 10.1371/journal.pone.0137317

**Published:** 2015-09-23

**Authors:** W. Apoutou N'Djin, Jean-Yves Chapelon, David Melodelima

**Affiliations:** 1 LabTAU, Inserm U1032, Institut National de la Santé et de la Recherche Médicale, Lyon, France; 2 Université Claude Bernard Lyon 1, Lyon, France; Okayama University, JAPAN

## Abstract

Organ motion is a key component in the treatment of abdominal tumors by High Intensity Focused Ultrasound (HIFU), since it may influence the safety, efficacy and treatment time. Here we report the development in a porcine model of an Ultrasound (US) image-based dynamic fusion modeling method for predicting the effect of *in vivo* motion on intraoperative HIFU treatments performed in the liver in conjunction with surgery. A speckle tracking method was used on US images to quantify in vivo liver motions occurring intraoperatively during breathing and apnea. A fusion modeling of HIFU treatments was implemented by merging dynamic *in vivo* motion data in a numerical modeling of HIFU treatments. Two HIFU strategies were studied: a spherical focusing delivering 49 juxtapositions of 5-second HIFU exposures and a toroidal focusing using 1 single 40-second HIFU exposure. Liver motions during breathing were spatially homogenous and could be approximated to a rigid motion mainly encountered in the cranial-caudal direction (f = 0.20Hz, magnitude >13mm). Elastic liver motions due to cardiovascular activity, although negligible, were detectable near millimeter-wide sus-hepatic veins (f = 0.96Hz, magnitude <1mm). The fusion modeling quantified the deleterious effects of respiratory motions on the size and homogeneity of a standard “cigar-shaped” millimetric lesion usually predicted after a 5-second single spherical HIFU exposure in stationary tissues (Dice Similarity Coefficient: DSC<45%). This method assessed the ability to enlarge HIFU ablations during respiration, either by juxtaposing “cigar-shaped” lesions with spherical HIFU exposures, or by generating one large single lesion with toroidal HIFU exposures (DSC>75%). Fusion modeling predictions were preliminarily validated *in vivo* and showed the potential of using a long-duration toroidal HIFU exposure to accelerate the ablation process during breathing (from 0.5 to 6 cm^3^·min^-1^). To improve HIFU treatment control, dynamic fusion modeling may be interesting for assessing numerically focusing strategies and motion compensation techniques in more realistic conditions.

## Introduction

Treatment safety and accuracy when using High Intensity Focused Ultrasound (HIFU) can be challenged by organ motions, particularly during breathing [1,2,3,4]. Periodic deformations and motions of the lungs during breathing [5] are transmitted to surrounding structures and can significantly modify the position of abdominal organs, depending on their location, anatomical structure, tissue consistency and/or amount of freedom of movement within the body. For liver HIFU treatment applications, respiratory motion transmission through the diaphragm is critical and can range from millimeters to dozens of millimeters [6,7,8,9,10]. In order to avoid liver motions from interfering with treatment targeting and monitoring, some previous preclinical studies have highlighted the interest of using a “breath-hold” protocol by applying intermittent apnea during HIFU exposures [11,12,13]. Intermittent apnea was well tolerated by animals and was found to be convenient for ensuring accurately targeted treatment, but this approach could present clinical limits. In surgery (resection) and in procedures involving thermal ablation with physical agents, localized tumors must be ablated entirely with safety negative margins in order to ensure treatment efficacy. These margins are critical to prevent the risk of local recurrence and were shown to increase overall patient survival rates [14,15]. Standard margins in surgery and radiofrequency can range from 1 to 20 mm in all directions depending on the tumor size, location and amount of free space in the liver [16,17,18]. Since liver tumors can reach several centimeters in diameter, achieving HIFU ablations with negative margins implies repeating/extending HIFU exposures, which could complicate the “breath-hold” strategy. In addition, repetition and frequency of induced artificial apnea can lead to hemodynamic disorders [19,20]. Therefore, allowing respiratory activity during HIFU treatments may be important for optimizing treatment performances, safety and enhancing post-treatment recovery.

To eliminate the adverse effects associated with respiration which penalize both HIFU targeting accuracy and its monitoring, several studies have proposed strategies using Magnetic Resonance Image (MRI) guidance for real-time compensation of organ motions. On the monitoring side, several MR acquisition approaches have been described, either to synchronize acquisitions on periodic motions (gating) [21], to track the motion (navigator echoes) [22,23] or to correct complex MR data (Multibaseline or referenceless acquisitions) [24,25,26,27] for allowing precise MR temperature monitoring in abdominal organs during breathing. On the therapy side, methods have been reported to improve HIFU performances during breathing, either with motion gating and intermittent HIFU exposures, or with motion tracking, by correcting the position of the focal zone using ultrasound beam steering with phased-array transducers [28]. The organ motion was then compensated without physical displacement of the transducer. These techniques have been proposed in order to demonstrate the ability to control heat deposition during extracorporeal procedures using conventional highly focused spherical HIFU transducers. However, extracorporeal generation of large thermal ablations during breathing remains challenging, as it requires delivering sufficient ultrasound energy into the liver through multiple attenuating tissue layers, and the rib cage acting as an acoustic barrier. Ultrasound beam steering strategies for motion compensation and ablation extension are also associated with limited spatial windows of focus deflection, which depend on the characteristics of the phased array transducers used.

As an alternative approach for treating abdominal diseases, intraoperative HIFU has proven promising for providing a complementary tool for open surgery. This intraoperative approach provides ideal conditions for generating large and fast HIFU treatments in the liver, and then assist surgery with appropriate HIFU exposures [29,30,31]. Ultrasound (US) imaging is a standard method for guiding interventional therapies in real-time and US-guided HIFU techniques (USgHIFU) have already proven successful in clinical environments for various applications [32,33,34,35,36,37,38]. In our team, an intraoperative USgHIFU strategy has been developed by proposing a concept of toroidal ultrasound focusing. It has been demonstrated that the extended focal zone of a toroidal-shaped HIFU transducer associated with appropriate exposure parameters enables fast generation of large single lesions in the liver during an open surgical procedure (5–8 cm^3^ in 40s) [13,39]. An experimental therapeutic system dedicated to the treatment of Liver Metastasis from Colorectal Cancer (LMCC) has been validated at preclinical level in a porcine model [40,41]. More recently, an evolution of the toroidal focusing strategy demonstrated potential for increasing lesion volume [42].

Optimization of HIFU strategies for *in vivo* treatment during breathing, however, requires accurate prediction and quantification of the effects induced by organ motions on HIFU lesions in realistic conditions. Previous studies have already highlighted *in vitro* effects of artificial and approximate liver motions on HIFU treatments [1]. However, real *in vivo* liver motions are more complex [3,4] and may lead to various effects on induced lesions. Because of the difficulty in precisely accessing lesion volumes *in vivo*, there is no quantitative description of the effect of *in vivo* liver motion on HIFU treatments. In the literature, few data are available regarding the ability to generate large thermal ablations accurately and achieve treatment volumes which are compatible with liver metastasis ablation. To access information regarding the lesion, numerical modeling techniques may be advantageous in conjunction with imaging techniques, as together they allow quantification of multiple parameters in 4D with optimal spatial and temporal resolutions. HIFU modeling techniques are well established to simulate thermal effects in stationary homogeneous tissues and are now essential for designing HIFU transducers and planning *in vitro* treatment outcomes [43,44,45,46]. To date, however, HIFU numerical modeling suffers from a lack of *in vivo* validations in realistic conditions. An accurate consideration of experimental conditions remains indeed very challenging for developing realistic simulations, and would ideally require performing *in vivo* measurements of all physiological and biomechanical parameters at the moment of the interventional procedure.

The present paper presents a US image-based dynamic fusion modeling method which enables numerical modeling of the effects of *in vivo* real liver motions on the size, shape and location of thermal lesions induced during intraoperative USgHIFU treatments. The aim of this work was first to develop a hybrid method incorporating dynamically real and modeled ultrasound data for studying HIFU ablations in moving tissues, which could be implemented with standard US imaging systems available in clinical settings. This required studying the nature of *in vivo* liver tissue motion in the conditions of intraoperative USgHIFU procedures. The second objective was to show the potential of US image-based dynamic fusion modeling technique to compare the performances of various USgHIFU strategies, by showing quantitative and consistent information could be provided using this method in establishing HIFU lesions in *in vivo* liver tissues during respiration. The reliability of the proposed fusion modeling method was then preliminarily validated *in vivo* and discussed for 2 HIFU exposure strategies (multiple-short and single-long HIFU exposures) performed with 2 different shapes of HIFU transducers (spherical and toroidal).

## Materials and Methods

### Fusion modeling of HIFU treatments: principle

**Fig 1:**
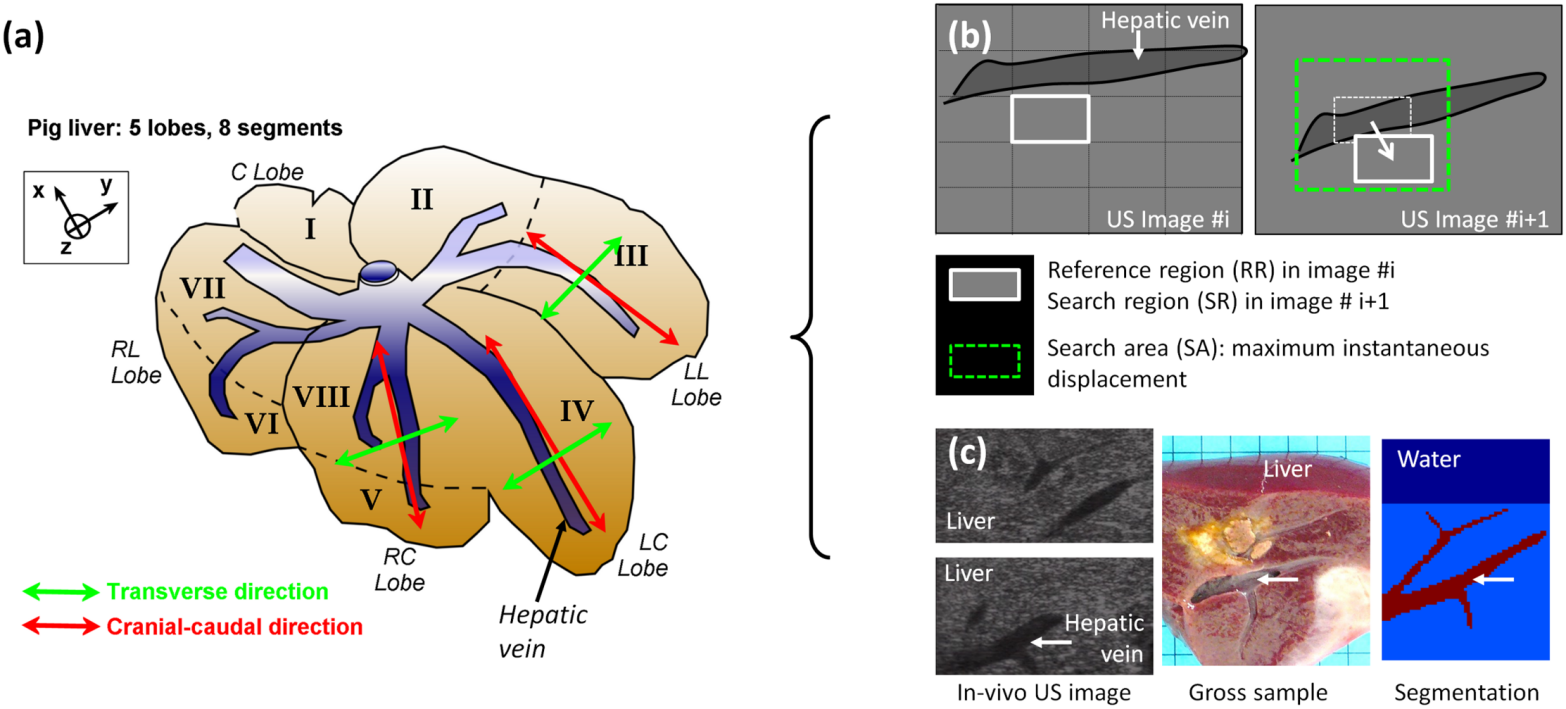
US-image based dynamic fusion modeling: principle. **(a)** Position of the ultrasound imaging probe during acquisitions of *in vivo* liver motion in a porcine model (green and red arrows). Ultrasound sequences were acquired in Left Lateral (LL), Left Central (LC) and Right Central (RC) lobes of the liver, as well as in 2 orthogonal planes (cranial-caudal and transverse); **(b)** Estimation of tissue motion using a speckle tracking method applied on B-mode ultrasound images: principle; **(c)** Tissue segmentation on anatomical ultrasound images: performed post-operatively in this study and assisted by macroscopic observations of gross sample. Dynamic fusion modeling of HIFU treatments was performed by combining real data of *in vivo* tissue motions and anatomical structures with simulated HIFU data.

The principle of the fusion modeling method introduced here is to fusion data from various sources: real and virtual, static and dynamic. In this study, fusion modeling was implemented by carrying out an *in vivo* study on intraoperative liver HIFU treatments and by including the following steps: (i) Ultrasound image acquisition of *in vivo* liver motions in a porcine model, (ii) Estimation of tissue motion using ultrasound speckle tracking on B-mode images, (iii) Tissue segmentation on anatomical ultrasound images, (iv) Fusion between dynamic *in vivo* and simulated data (Fig 1). These steps are detailed in the following sections.

### Ethics statement

All animal experiments were carried out in strict accordance with the legal conditions of the French National Ethics Committee for Reflection on Animal Experimentation (CNREEA) and the presented study was approved by the local Ethics Committee on Animal Experiments of the Institute of Experimental Surgery (ICE, Léon Bérard Centre, Lyon, France; CNREEA Code: C2EA-10). To minimize the number of animals undergoing surgical procedures, initial investigations carried out for studying pig liver movements, requiring intraoperative acquisition of ultrasound images only (no HIFU), were performed during an ongoing *in vivo* study. In this study, HIFU exposures were already planned during an open procedure for studying an intraoperative USgHIFU system dedicated to LMCC treatment [47]. All *in vivo* investigations were performed in a porcine model (Landrace). To minimize animal suffering, pigs were anesthetized, mechanically ventilated during the intervention, monitored, and then sacrificed according to the ICE standard surgical protocol as described in previous studies [41].

### Ultrasound image acquisitions of *in vivo* liver motions

Ultrasound imaging sequences were acquired *in vivo* in the liver of 4 healthy pigs with an average weight of 27 ± 4 kg (range 22–31 kg). All intraoperative USgHIFU procedures were carried out after a 25 cm median laparotomy performed using the xiphoid process (lower part of the sternum). Oxygenation was supplied from an assisted ventilation system (ABT 4300, Kontron Instruments) at a rate of 7.2 l·min^-1^ and a frequency of 12 cycles·min^-1^ (duty cycle: 40%). Liver motion acquisitions were performed during periods of breathing and periods of artificial apnea created by temporally interrupting mechanical ventilation for a maximum of 2.5 minutes, while maintaining a minimum oxygen saturation of 80%. The ultrasound scanner used was a BK® HAWK 2102 EXL (B-K Medical, Herlev, Denmark). Ultrasound image acquisitions were performed using a 12 MHz linear array ultrasound imaging probe with 63% fractional bandwidth (Model 8805, B-K Medical, Denmark). Images were acquired under the same conditions and orientations as those seen during USgHIFU exposures in liver. The ultrasound imaging probe was placed in acoustic contact with the superior face of the liver. The static pressure applied on the liver surface with the ultrasound device did not suppress respiratory motion and was accounted for in our analyses to estimate the motion effect in realistic conditions of intraoperative HIFU treatments. The field-of-view (FOV) of the ultrasound image was 45 mm (deep) by 27 mm (wide) and the frame rate was 54 fps.

### Determination of the nature of *in vivo* liver movements by ultrasound

Ultrasound imaging sequences were performed on the first 2 pigs during breathing, in the left lateral (LL), left central (LC) and right central (RC) lobes of each pig. In each location, 2D US images were acquired in 2 orthogonal planes: i) along the cranial-caudal direction which corresponded to the main liver motion observable by the surgeon when visually following the liver surface during animal respiration; and ii) in a direction transverse to the main motion (Fig 1a). The rationale was the necessity to quantitatively verify that liver motion could be approximated to a 2D motion and assess the suitability of 2D-US imaging techniques for implementing 3D fusion modeling methods in the context of intraoperative USgHIFU in the liver. The right lateral (RL) and caudate lobes (C) were not observed as they were less accessible in the abdomen and did not allow acquisition of 2 orthogonal planes with the ultrasound imaging probe. Ultrasound images were acquired in the remaining 2 pigs in regions containing sus-hepatic veins in order to determine the relative influence of respiration and cardiovascular activity on liver motions. First of all, acquisitions were obtained during breathing to access liver movement caused by respiration. Secondly, acquisitions were performed during apnea to separate the liver movement caused only by cardiovascular activity. Special attention was focused on liver tissues surrounding sus-hepatic veins, while veins were dilated with blood pressure pulses.

Following that, an ultrasound correlation-based method was used to estimate *in vivo* liver motion using speckle tracking on B-mode signal. This method has already showed better results than methods using the radio frequency (RF) signal, especially when the deformation or the out-of-plane-motion of the observed feature leads to large decorrelation [48]. The 2D motion tracking algorithm used in the presented study was previously described by Hsu *et al*. (2005) [49], validated *in vivo* in 3D by Harris *et al*. (2010) [10], and will be covered here only briefly. The tracking algorithm estimated the displacements of a specific region between 2 images by selecting a reference region (RR) in a first image, and by determining in the consecutive image the location of the search region (SR) exhibiting the most similar speckle pattern (Fig 1b). A pixel-by-pixel cross-correlation coefficient, ρ_AB_, between two such regions, A and B, was calculated as follows:

$$\rho_{AB}=\frac{\sum_{i=0}^{N-1}\left[a(i)-\bar{A}\right]\left[b(i)-\bar{B}\right]}{\sqrt{{\sum_{j=0}^{N-1}\left[a(j)-\bar{A}\right]}^{2}{\sum_{k=0}^{N-1}\left[b(k)-\bar{B}\right]}^{2}}}$$ (1)

with N being the number of pixels contained in each region, and a and b the discrete grey levels constituting the speckle patterns of regions A and B respectively. The SR was selected within a predetermined search area (SA) and translated in either the axial or lateral directions in relation to the US image. Cross-correlation coefficients were calculated for all possible positions of SRs in order to cover the entire SA. The hypothesis of a RR associated with a unique US speckle pattern was made in order to consider the location of the SR showing maximum correlation as the new position of the RR in the consecutive image. Subpixel estimations of the displacement could be made by interpolation between the position of the maximum correlation coefficient and those of the neighboring correlation coefficients. This process was then repeated in a piecewise manner for multiple RRs and over the entire image acquisition, in such a way that all displacements inside a region-of-interest (ROI), typically the whole US image truncated with margins equal to SA dimensions, could be estimated. The tracking program generated two displacement maps which contained data of in-plane longitudinal and transverse displacements, oriented respectively along, and perpendicularly to, the ultrasound propagation direction. A third map was created containing correlation coefficients between RRs over time. Space and time averaged correlation coefficients were calculated for assessing the quality of the motion detection in different liver regions (LL, LC and RC lobes) and for 2 imaging orientations (cranial-caudal and transverse). The US speckle tracking parameters were optimized manually for each case based on the reconstructed motion curves. Saturation patterns could typically arise if the maximum tissue motion was missed by the tracking procedure (if the displacement between 2 images is larger than the SR), leading to measurement errors and systemic drifts in the total motion detected over time. Another cause of distortions in the motion curves could be due to mistracking. Thus the RR and SR windows were sized to emphasize the unicity of the speckle pattern of the regions selected in US images, perform a sufficiently large research scan to cover the maximum instantaneous tissue displacement expected between 2 US images, and maximize the correlation coefficients inherent to the tracking process. To achieve full detection of the motion magnitude, the SR window was delineated based on the RR window at which additional search margins were added in all directions. Those search margins were chosen to be greater than the maximum expected displacement of the RR window between two successive US images. The overlapping of RR windows was adjusted in the cranial-caudal and transverse dimensions to control the spatial sampling of the displacement maps. By default, tracking parameters were set to compute 2D displacement maps with a submillimeter scale spatial resolution for studying any complex elastic tissue motions. These parameters were optimized to decrease the computational time: a truncated US image was used for focusing the tracking on a reduced FOV, the US image sampling time was minimized and for statistical analysis, a global average tissue displacement was tracked when an approximation to a rigid motion could be justified by preliminary observations. All tracking parameters have been summarized in Table 1.

**Table 1:** US-speckle tracking key parameters for *in vivo* liver motion detection in a porcine model.

**Spatial sampling of the US image (pixel/mm)**	9.6					
**Sampling time (image/s)**	25					
**US image total FOV (mm)**	27.0 (Lateral, CC / T), 45.0 (Depth, AP)					
**US image truncated FOV for tracking (mm)**	27.0 (Lateral, CC / T), 18.5 (Depth, AP)					
**Measurement conditions**	Apnea		Respiratory activity			
**Liver motion model**	Elastic		Rigid			
**Measured data**	2D map of the motion		2D map of the motion		Average motion	
**Anatomical orientation**	CC / T	AP	CC / T	AP	CC / T	AP
**RR, SR (mm)**	4.1	1.0	10.3	4.1	10.3	4.1
**SA (mm)**	5.1	2.1	16.4	6.2	16.4	6.2
**ROI (mm)**	21.9	16.4	10.6	12.3	10.6	12.3
**Maximum tissue motion magnitude detectable (mm)**	2.6	1.0	8.2	3.1	8.2	3.1
**Maximum tissue speed detectable (cm/s)**	6.4	2.6	20.5	7.8	20.5	7.8
**RRs overlapping (mm)**	3.6 (88%)	0.9 (90%)	9.7 (95%)	40 (98%)	_	
**Spatial sampling of the displacement map (pixel/mm)**	1.9	9.6	1.9	9.6	_	

**FOV**: Field Of View; **RR**: Reference Region; **SR**: Search Region; **SA**: Search Area; **ROI**: Region Of Interest; **CC**: Cranial-caudal direction; **AP**: Anterior-Posterior; **T**: Transverse

### Fusion between dynamic 2D US speckle tracking data and a 3D numerical modeling of HIFU treatment

A dynamic fusion modeling method was implemented to simulate realistic *in vivo* intraoperative HIFU ablations in moving liver tissues. *In vivo* data obtained from US-speckle tracking and tissue segmentation were integrated within a 3D finite element numerical modeling tool previously described for simulating HIFU treatments in stationary biological tissues [44].

For this method, the spatial distribution of the acoustic pressure field is calculated using the Rayleigh surface integral, while including a calculation of the global harmonic attenuation of pressure, to account for non-linear effects on ultrasound propagation arising from stable oscillations of microbubbles present in biological tissues during HIFU treatments [44,45]. In this model, the Gilmore-Akulichev equation is used to estimate the distribution of the harmonic frequencies contained in the acoustic pressure scattered by the microbubbles within tissues [50]. The absorbed ultrasound energy deposited in tissue, Q, was then estimated by accounting for the absorption of the incident wave at the fundamental frequency and the stronger absorption of the high-frequency harmonics created by microbubbles:

$$Q=\beta \frac{p^{2}}{2\rho_{t}cV}(1-e^{-2A_{Harm}V})$$ (2)

where p is the acoustic pressure, c is the speed of sound, V is the volume of attenuating tissue, and A_Harm_ is the global harmonic attenuation which represents the attenuation of all harmonics scattered by microbubbles with an initial radius R_0_ and subjected to acoustic pressure p. For given R_0_ and p, A_Harm_ is a function of the radial distance r and the density of microbubbles present in tissues N_μbb_. The coefficient β ≤ 1 represents the ratio of ultrasound energy actually absorbed per unit volume. When lower than unity, β expresses the fact that acoustic attenuation is not only due to absorption α, as scattering is also included. The temperature increase induced by HIFU exposures in the liver is then estimated by solving the Bio Heat Transfer Equation [43,51]:

$$\rho_{t}C_{t}\frac{\partial T}{\partial t}=k\Delta T-\omega_{b}C_{b}(T-T_{b})+Q$$ (3)

where ρ_t_ is the density of tissues, C_t_ is the specific heat capacity of such tissues, T is tissue temperature, t is time, k is thermal conductivity, ω_b_ is blood perfusion coefficient, C_b_ is specific heat capacity of the blood, T_b_ is blood temperature (37°C in *in vivo* conditions, body temperature at the equilibrium) and Q the absorbed ultrasound energy deposited as heat source in tissues.

Based on this model validated in stationary biological tissues, a 3D numerical modeling of HIFU treatments in dynamic tissues was implemented by integrating data of liver displacements measured by the 2D motion tracking method. Then, the calculation of heat spatial distribution during simulated ultrasound exposures was modified to account for liver tissue displacements. For the fusion of *in vivo* motion data in the 3D numerical tissue grid, the Ox transverse axis was chosen to lie in the cranial-caudal direction, the Oy transverse axis was defined in the transverse direction, and the Oz longitudinal axis was normal to the surface of the liver (anterior-posterior orientation, liver tissue depth). When considering the nature of liver movements quantitatively, it appears that the overall motion can reasonably be approximated to a nonlinear motion with a main component lying in the cranial-caudal direction. Then, integrating motion data from 2D ultrasound images acquired in a sagittal plan including the cranial-caudal direction enabled the modeling of most phenomena. Although tissues expansions and contractions were detectable in all dimensions (Ox, Oy and Oz) using the motion tracking method [49], initial analysis of the liver motions confirmed that the major displacements occurring during respiration were homogeneous in ROIs with sizes ranging in accordance with those of HIFU focal regions (millimeters to centimeters). Then, modeling liver tissue motion in 3D as a 2D rigid motion along the sagittal plan (xOz) was considered acceptable as a first approximation to estimate *in vivo* respiratory motion effect on the creation of HIFU lesions. To account for liver displacements in the model, elementary heat dQ_breath_(x,y,z,t) deposited during a time dt to an elementary volume dV located at a point M(x,y,z) was expressed as follows:

$$dQ_{breath}(x,y,z,t)=\{\begin{array}{ll} dQ\;(x-dx(x,z,t), & \\ \;y,z-dz(x,z,t),t), & for\;x-dx(x,z,t)\in \left[x_{\min},\;x_{\max}\right]\\ & and\;z-dz(x,z,t)\in \left[z_{\min},\;z_{\max}\right].\\ 0, & elsewhere. \end{array}$$ (4)

where dQ_breath_(x,y,z,t) is the map of elementary heat deposited in moving tissues, dQ(x,y,z,t) is the map of elementary heat deposited in stationary tissues, dx(x,z,t) and dz(x,z,t) are the tissue displacement maps respectively along transverse and longitudinal axes (perpendicular and parallel to the ultrasound propagation direction), x_min_, z_min_, x_max_ and z_max_ are the coordinates of the map extremities. The map of elementary heat, dQ, was then replaced by dQ_breath_ for solving the BHTE. Finally, the model of thermal dose based on the equivalent time at 43°C (t_43°C_) was used to estimate numerically thermal damages induced by HIFU exposures in the liver:

$$t_{43{}^{\circ}C}(t)=\int_{0}^{t}R^{43-T(t)}dt,\;R=\{\begin{array}{l} 0\;T<37{}^{\circ}C\\ 0.25\;T\in \left[37{}^{\circ}C;\;43{}^{\circ}C\right]\\ 0.5\;T>43{}^{\circ}C \end{array}$$ (5)

where t_43°C_ is the thermal dose in Cumulative Equivalent Minutes (CEM), T is current tissue temperature and t is time [52]. A minimum threshold for irreversible damage (t_43°C_ref_) was set to a commonly accepted value of 240 CEM to provide a conservative predictor of the extent of severe thermal lesions in liver tissues, according to hyperthermia and HIFU literature in various soft tissue types [45,53,54,55,56]. All physiological and acoustical parameters used to model liver tissues are summarized in Table 2 [57,58,59,60,61]. Liver tissues were simulated as dynamic regions moving with respiration, which could exhibit either homogenous or inhomogeneous physiological properties, depending on the presence of sus-hepatic veins. For this last configuration, manual segmentations of hepatic veins were performed using *in vivo* ultrasound images acquired intraoperatively (12 MHz imaging probe). Sus-hepatic main branches could be estimated in size and shape by scanning the region of interest with 2D US images longitudinally and transversally. Post-operative segmentations of the main sus-hepatic structures on US images were also assisted with direct anatomical observations performed *in vivo* during macroscopic analyses on the dissected liver. The interaction between the sus-hepatic vein and HIFU exposure actions was modeled by assuming that blood heating, which could arise from HIFU energy absorption in blood or heating diffusion from liver tissues to blood, was negligible compared to the energy dissipated by the blood circulation. Thus, in simulation, the blood temperature in large sus-hepatic veins (> 1 mm in diameter) was considered constant during HIFU exposures and was fixed at 37°C, as previously proposed for HIFU modeling studies in cardiac applications [57]. The effect of the HIFU lesion on the liver microperfusion was accounted for by cancelling the perfusion parameters in tissue regions exhibiting irreversible damage (t_43°C_ ≥ t_43°C_ref_). Variations of the acoustic properties of tissues which may arise due to thermal or biological changes during HIFU exposures were not taken into account in this study.

**Table 2:** Liver tissue physiological parameters used during numerical modeling of HIFU treatments.

Soft tissue density, ρ_t_ (kg∙m^-3^)	1060	[45,58]
Acoustic absorption, α (Np∙m^-1^·MHz^-1^)	2.6	[58,59]
Acoustic attenuation in vivo, A (Np∙m^-1^·MHz^-1^)	4.5 (β = 0.6)	[58]
Speed of sound, c (m∙s^-1^)	1540	[58]
Initial tissue temperature at the equilibrium, T_0_ (°C)	37	
Thermal conductivity of soft tissues, k (W∙m^-1^°C^-1^)	0.5	[45,58,60]
Specific heat capacity of soft tissue, C_t_ (J∙kg^-1^°C^-1^)	3700	[45,58,61]
Blood perfusion in tissues, ω_b_ (kg·m^-3^·s^-1^)	30 (t_43°C_ < t_43°C_ref_); 0 (t_43°C_ ≥ t_43°C_ref_)	[45,58]
Blood temperature in sus-hepatic veins, T_b_ (°C)	37	[57]
Specific heat capacity of blood, C_b_ (J∙kg^-1^°C^-1^)	3770	[45,58]
Density of microbubbles in tissues, N_μbb_ (μbubbles∙mm^-3^)	200	[44]
Initial radii of the bubbles, R_0_ (μm)	1–3	[44]
Minimal thermal dose threshold for irreversible damage in soft tissues, t_43°C_ref_ (CEM)	240	[45,53,54,55,56]

### Dynamic fusion modeling for estimating the effect of liver motion on 2 intraoperative HIFU focusing strategies: spherical and toroidal

**Fig 2:**
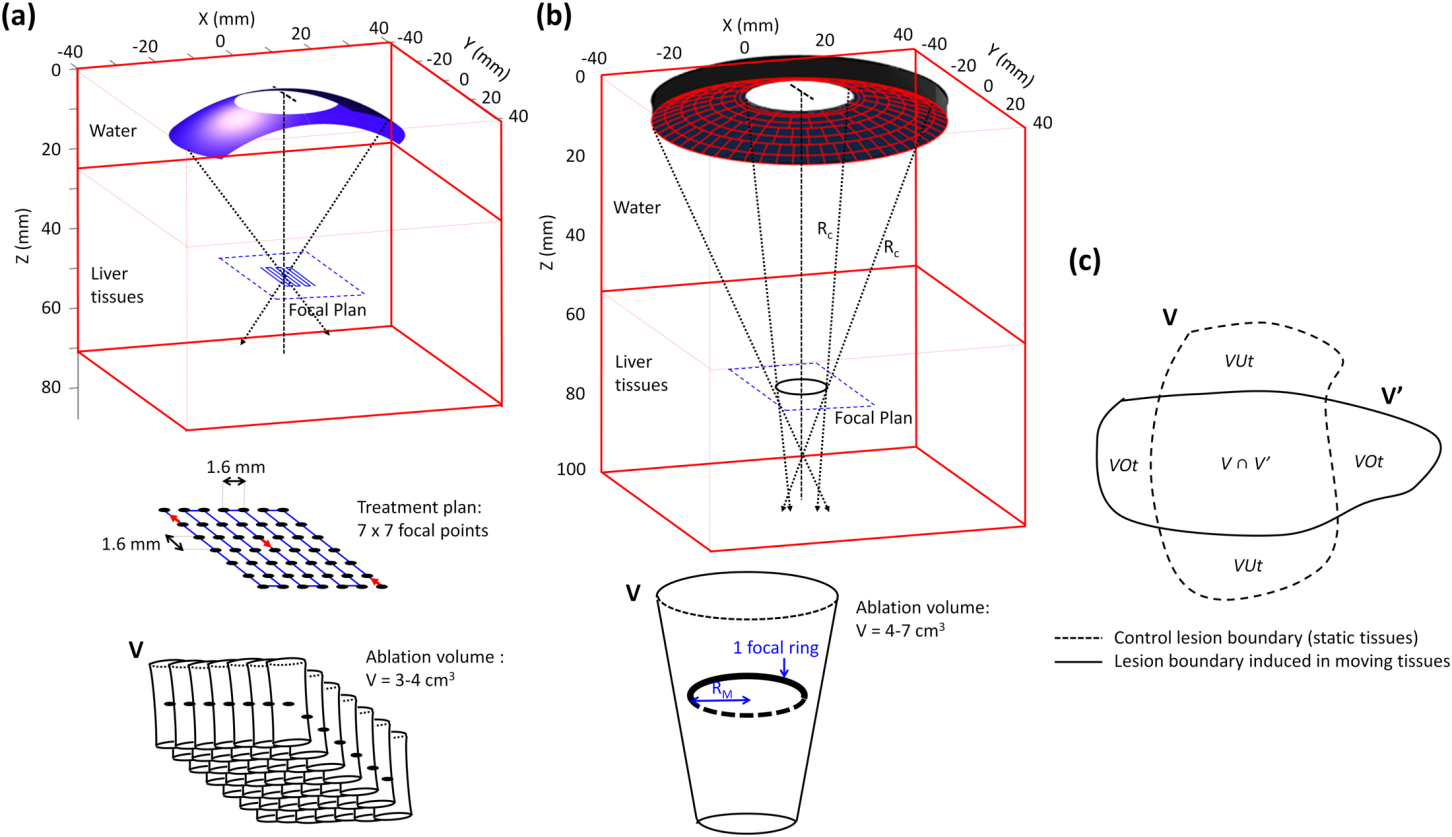
Quantitative impact of *in vivo* tissue motion on 2 different HIFU focusing strategies. **(a)** A spherical HIFU focusing: 7 x7 millimetric cigar-shaped lesions; Pac = 30W, HIFU sequence: 5s ON / 5s OFF, total treatment time = 8 min; **(b)** a toroidal HIFU focusing: 1 single centimetric lesion, Pac = 50W, HIFU sequence: 40s ON, total treatment time = 40 s; **(c)** Schematic illustrating the different volume measurements performed for quantifying the effect of tissue motions on HIFU lesions, with V being the volume of the Control lesion (without motion), V’ the volume of lesion when induced in moving tissues, VUt the volumetric undertreatment and VOt the Volumetric Overtreatment.

Simulations were carried out for 2 HIFU medical devices working at a 3 MHz frequency, but associated with 2 different strategies of HIFU focusing and treatment planning: spherical focusing delivering multiple juxtapositions of short-duration HIFU exposures and a toroidal focusing associated with a single, long-duration HIFU exposure [29,40]. The first simulated device was a mono-element truncated spherical transducer previously used for preliminary investigations on liver HIFU ablation (radius of curvature R_c_: 45 mm, transducer aperture in the cranial-caudal direction: 56 mm, truncation of the aperture in the transverse direction: 33 mm) [29]. The second simulated device was a 256-element phased-array toroidal HIFU transducer which has been specifically developed at the preclinical level for LMCC treatments (geometric model: spindle torus, “minor radius” of the torus or radius of curvature R_c_: 70 mm, “major radius” of the torus or distance between the axis of revolution and the center of the circle generating the radius of curvature R_M_: 5 mm, aspect ratio R_M_/R_c_: 0.07, transducer aperture: 68 mm) [13,39]. Both HIFU transducers were simulated with a circular hole at their centers (diameter: 25 mm) to account for the presence of ultrasound imaging probes integrated within the existing HIFU medical prototypes. First, the effects of liver motions were studied with the spherical transducer for a millimeter scale HIFU lesion created with a single, short-duration exposure (HIFU exposure sequence: 5s On, acoustic power: 30 W, geometry of the focal zone at -6 dB of maximum pressure: ellipsoid, minor/major widths: 0.5/0.7 mm in the focal plan, focal length: 3.5 mm, acoustic intensity at the focal point: I_SATA_ = 5000 W·cm^-2^ in water, 3800 W·cm^-2^ in tissues for a transducer-to-tissue distance d_t-t_ = R_c_-10 mm). Second, liver motion effects were analyzed in the case of a larger HIFU treatment performed within ~8 minutes after juxtapositions of 49 single HIFU lesions (49 exposures of 5s On / 5s Off for each), as described in a previous study [29]. The HIFU exposures included 7 x 7 juxtaposed millimetric ellipsoidal HIFU lesions, each separated by a 1.6-mm step (Fig 2a). This exposure plan was chosen to generate a necrosis comparable in volume to a large single lesion obtained with the toroidal HIFU device. Third, liver motion effects were studied on a large single HIFU lesion generated with the toroidal HIFU transducer (exposure time: 40s On, acoustic power: 60 W). The focusing parameters and the HIFU exposure sequence were optimized for generating a large conical lesion of 4–7 cm^3^ in less than 1 minute (Fig 2b). In this study, the 256 elements of the phased-array toroidal transducer were driven in phase. The first geometric focal distance of the toroidal transducer, determined by the “minor radius” of the spindle torus (radius of curvature), was 7 cm. The geometry of the focal zone, a ring of 10 mm in diameter, 2 mm thick (-6dB of maximum pressure at the focal plan), was determined by the “major radius” of the spindle torus (acoustic intensity within the focal ring: I_SATA_ = 90 W·cm^-2^ in water, 70 W·cm^-2^ in tissues for d_t-t_ = R_c_-10 mm). A second geometric focal distance appeared at 8.6 cm from the transducer and was due to the crossing of the ultrasound beam after the focal ring (geometry of the focal zone in the focal plane: a disk of 0.7 mm in diameter, acoustic intensity within the focal point: I_SATA_ = 6300 W·cm^-2^ in water, I_SATA_ = 3200 W·cm^-2^ in tissues for d_t-t_ = R_c_-10 mm) [40].

For both HIFU devices, simulations were conducted considering: (i) no motion (Control); and (ii) *in vivo* motion during breathing. The influence of liver motion on the efficacy and targeting accuracy of 2 HIFU focusing strategies was studied with the following key parameters: lesion diameters and depths (mm), lesion volume (cm^3^), treatment rate (cm^3^·min^-1^), temperature distribution in liver tissues (°C), t_43°C_ distribution (CEM), homogeneity, volumetric similarity (%), volumetric over- and undertreatment (cm^3^ and %). The homogeneity of HIFU lesions was assessed if no untreated space appeared within the lesion volume. A dice similarity coefficient (DSC) was calculated for volumetric comparisons between Control lesions and lesions modeled during breathing. The DSC is an overlap measure relating to the Jaccard Index and was calculated as follows:

$$DSC(V,V^{\prime })\left[\%\right]=\frac{2\times V\cap V^{\prime }}{V+V^{\prime }}\times 100$$ (6)

with V and V’ the lesion volumes with no tissue motion and with respiratory motion. Volumetric overtreatment, VOt, due to tissue motion was defined as the volume of additional irreversible lesions (t_43°C_ ≥ t_43°C_ref_) induced in regions of tissues originally located outside the boundaries of the Control lesions. Similarly, the volumetric undertreatment, VUt, corresponded to the volume of tissue originally coagulated in the Control lesion, which was no longer irreversibly damaged (t_43°C_ < t_43°C_ref_) when accounting for *in vivo* tissue motions (Fig 2c). The percentage of volumetric overtreatment VOt% (respectively undertreatment VUt%), was given as the volume ratio between VOt (respectively VUt) and the volume V of the Control lesion. To provide a comprehensive quantification of the variations induced by liver tissue motion on irreversible HIFU lesions, the thermal dose t_43°C_ has been given as a function of t_43°C_ref_:

$$\begin{array}{ll} t_{43{}^{\circ}C}\left[CEM\right]=a\cdot t43 & with\;t43=10^{12}\cdot t_{43{}^{\circ}C\_ref}\left[CEM\right]\\ & and\;a\in {\mathbb{R}}^{+} \end{array}$$ (7)

### Preliminary *in vivo* validation of fusion modeling

**Fig 3:**
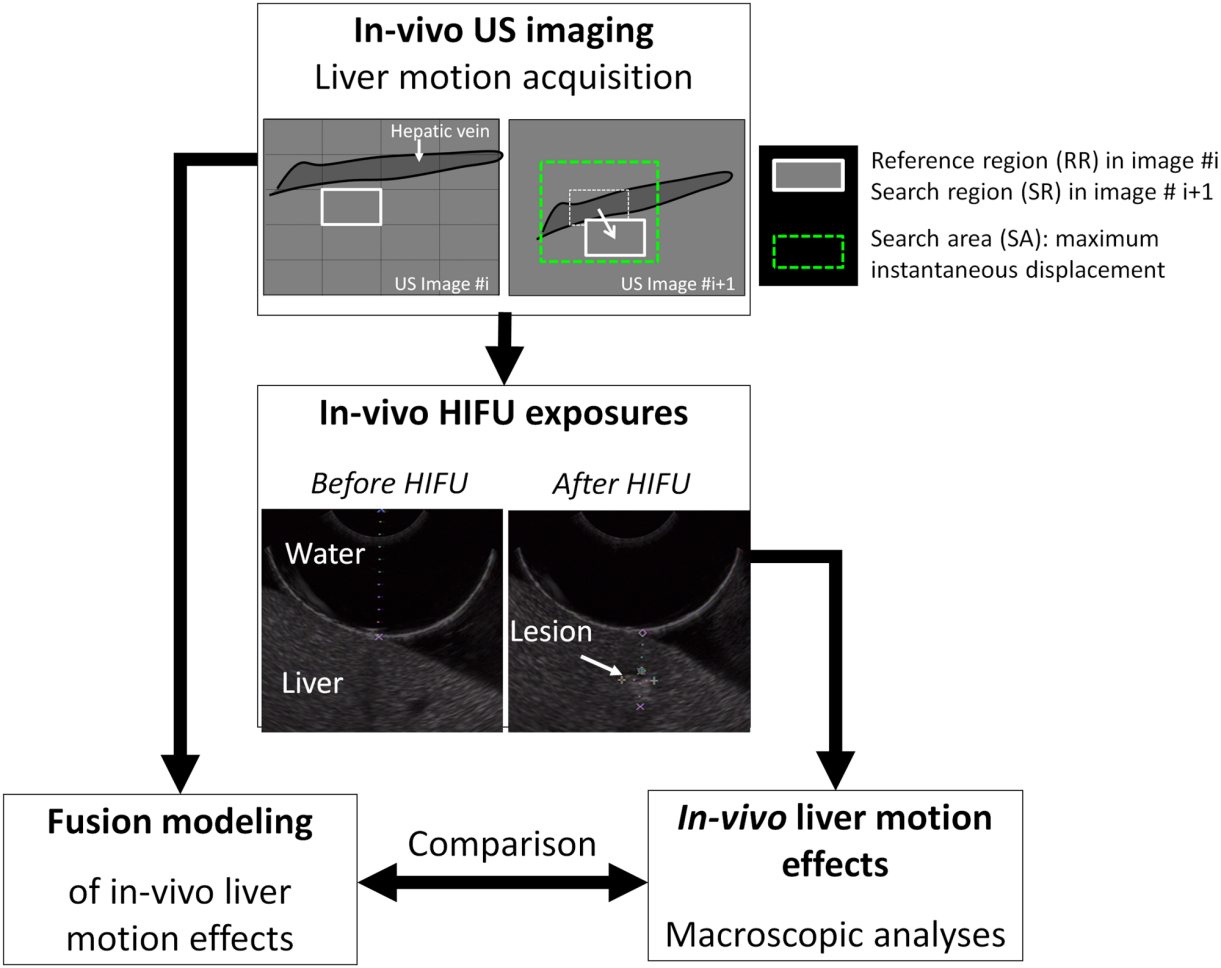
Study of a fusion modeling method for US-guided intraoperative liver HIFU ablations performed during respiration in a pig model. Methodology for preliminary validation of fusion modeling *in vivo*.

Here, the dynamic fusion modeling method was used to simulate in 3D real HIFU experiments performed *in vivo* in a porcine model with the spherical and toroidal HIFU systems described in the previous section. First, intraoperative HIFU treatments were performed *in vivo* in porcine liver during respiration. The emitting ultrasound transducers (therapy and imaging) were put into acoustic contact with the liver using an ultrasound coupling degassed fluid (Ablasonic®, EDAP, Vaux-en-Velin, France). The Ablasonic® was contained in a sterile polyurethane envelope (CIV-Flex Transducer cover, CIVCO, Kalona, IA), which also covered the devices, making it is possible to use the HIFU systems under sterile conditions. This sterile envelope attenuated the ultrasound pressure by about 2% at 3 MHz. A continuous flow (0.3 l/min) maintained the degassed coupling water at 20°C and enabled the cooling of the HIFU transducer during treatment. A peristaltic Masterflex pump (L/S model 7518–60, Cole-Parmer Instruments Co., Chicago, IL) drove the water around a closed-loop cooling circuit. The size of the cooling balloon at the front of the transducers was adjustable in such a way that various tissue depths could be targeted in the liver with the HIFU focal zone. The HIFU transducer-driving equipment was similar to that reported in previous studies [13]. The spherical HIFU probe was mounted on a motorized arm to scan the surface of the liver and complete the 7x7-lesion treatment plan, while the toroidal probe was held in place with a fixed mechanical arm. Just after completion of the HIFU exposures, ultrasound images of the ongoing tissue motions were acquired with the 12 MHz imaging probe. The HIFU-induced thermal lesions, which were associated with localized and highly contrasted changes in liver tissue echogenicity, were used as markers to guide US image acquisitions of tissue motions toward regions targeted with HIFU. In addition, they provided a clear reference region to be track on US images during respiration. Macroscopic examinations on gross samples were performed 14 days after the HIFU sessions for comparing fusion modeling estimations to *in vivo* HIFU lesions once they were completely established within the tissues (4–7 days after the treatment)[41]. Animals were followed up during this period (biological and clinical controls) and sacrificed under anesthesia at Day 14, following the anesthesia protocol described previously, to which was added an injection of 0.3 ml kg−1 embutramine, mebezonium iodine, and tetracaine hydrochloride (T61®, Intervet, France). After complete hepatectomy, the regions of the liver containing HIFU-induced coagulative necroses were sliced and removed while preserving a surrounding zone of native tissue. Macroscopic analyses were performed on these samples by cutting the lesions manually with a scalpel under ultrasound guidance (12 MHz imaging probe). With respect to the animal anatomy, the three axes of observation O*x*, O*y* and O*z* were respectively oriented in the cranial—caudal, left—right, and anterior—posterior directions. The cutting planes were determined using ultrasound image and after palpation of the sample to locate the HIFU lesion. The first cutting plan was always sagittal (xOz) to observe the effects of the main motion component on the shape of the HIFU lesion. A second plan was then observed in the axial direction (yOz, left-right) perpendicular to the main motion component. Liver motions encountered during *in vivo* HIFU sessions and estimated post-operatively with the US tracking technique at Day 0, were incorporated in the fusion modeling tool. The distance between the HIFU transducer and the anterior surface of the liver (size of the cooling balloon) as well as the thickness of liver lobes were reproduced in simulation based on data collected on US images. Main sus-hepatic branches (> 1 mm in diameter) present in the targeted liver regions were included in the model to account for realistic tissue structures in the ultrasound beam pathway. It also enabled consideration, along with the global tissue perfusion, of additional cooling effects arising locally by thermal diffusion due to large blood flows. Results of fusion modeling accounting for real *in vivo* liver motions were compared to real *in vivo* HIFU ablations carried out with the 2 HIFU devices (Fig 3). A prediction error will be considered if a significant difference of size, shape or homogeneity between lesions obtained experimentally and in modeling is observed. During *in vivo* experiments, HIFU exposures were not synchronized on the liver tissue motion, and the impact of the motion phase on the lesion deformation was not investigated here.

## Results

### Quantification of *in vivo* intraoperative liver movements with ultrasound imaging

The nature of *in vivo* liver tissue motions encountered during an intraoperative procedure was assessed from 2D US images acquired in LL, LC and RC liver lobes in the cranial-caudal and transverse plans. The performance of the 2D speckle tracking method was quantified *in vivo* with the cross-correlation coefficient calculated for each sequence. To ensure efficient motion tracking, the RRs and SRs were optimized manually based on the maximum amplitude and nature of tissue displacement. Respiratory motions, wider and of greater amplitude than motions caused by cardiovascular activity, required larger RR and SR for achieving maximal correlation in the tracking method. On the overall image sequences studied, the mean correlation coefficients in cranial-caudal and transverse plans were on average 0.79 ± 0.03 (range 0.75–0.81) and 0.67 ± 0.08 (range 0.58–0.79) respectively. The robustness of the speckle tracking was lower when measuring the transverse motion, since the main cranial-caudal motion was out of the image plan, penalizing the correlation between successive SRs. Measurement of the cranial-caudal motion was less affected by out of plan motions since transverse displacements have relatively low magnitudes. Speckle tracking decorrelation could result in measurement errors and significant drifts over time in the detection of cumulative motion. Correction coefficients could then be applied post-operatively to compensate for drifts and the relevance of the magnitude displacement detected was verified visually by comparing the displacement of a contrasted structure (lesion, hepatic vein) and that of an overlaid SR window moving virtually according to the tracking data on the US images.

**Fig 4:**
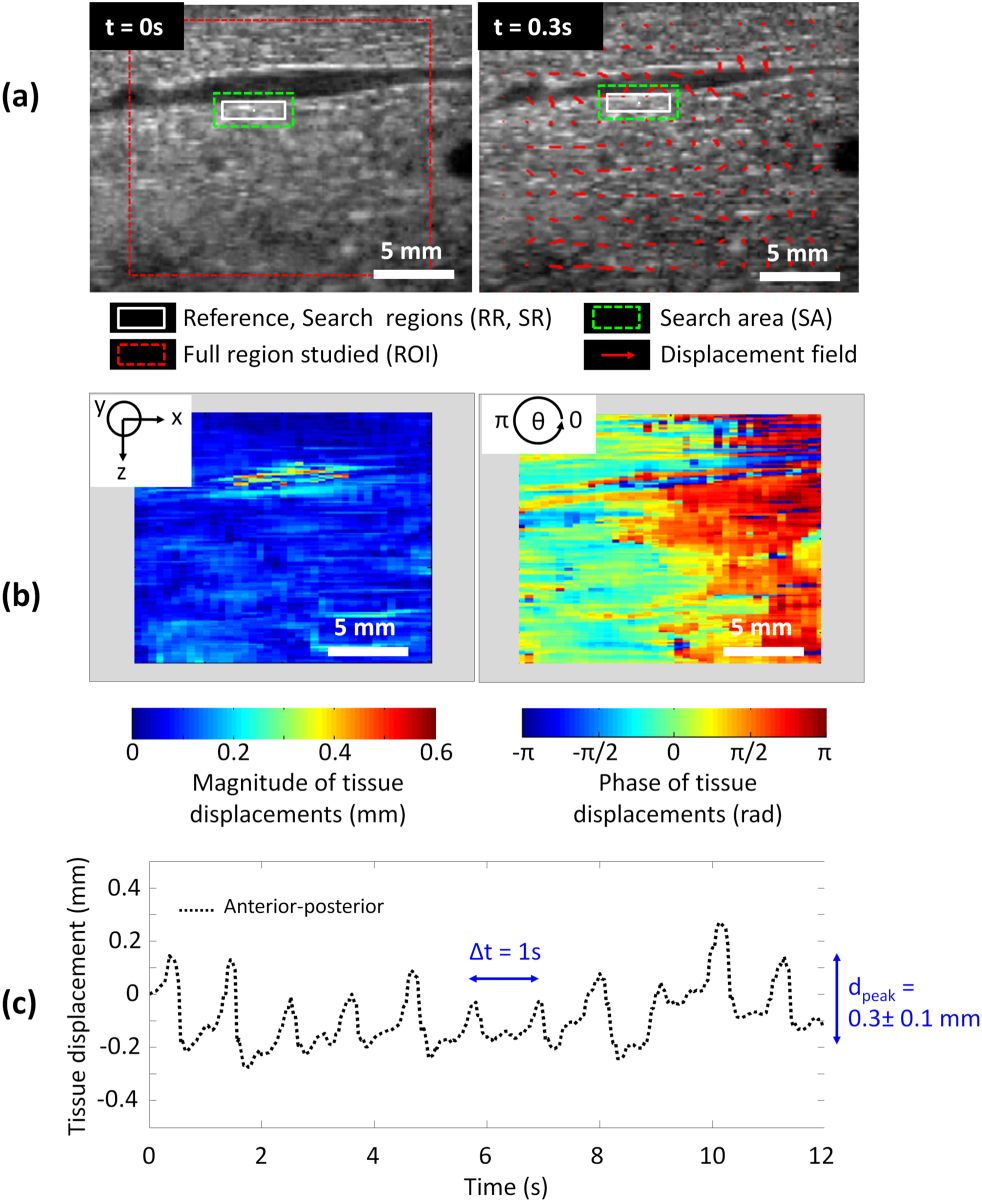
Submillimeter scale motion of a large millimeter-wide sus-hepatic vein during intermittent apnea induced artificially and detection of the cardiac activity. **(a)** 2D tracking of liver tissues at the boundary of the sus-hepatic vein for 2 different phases of the heartbeat cycle; **(b)** 2D spatial distribution of submillimeter scale elastic motion (anterior-posterior) around the sus-hepatic vein: magnitude and phase of the motion; **(c)** 1D motion component over time.

The US image speckle tracking was applied to homogenous regions of liver parenchyma and in regions of tissue including sus-hepatic veins which enabled study of the different sources of liver motion: a global tissue motion due to respiratory activity and a local motion induced by cardiovascular activity. In the experiments which included image acquisitions focused on sus-hepatic veins during apnea periods, the motion tracking method allowed direct access to the heartbeat frequency (f_heartbeat_ = 0.96 Hz) which was similar to the heartbeat frequency monitored with electrocardiogram (ECG monitoring: 56 beats/min, 0.93 Hz). The vein dilated and compressed periodically with blood pressure inflow locally inducing a submillimeter scale elastic tissue motion. The motion propagated mainly radially around the observed hepatic vein. This distribution was observable on US images acquired perpendicularly to the millimeter-wide vein (cross-section) and the predominance of radial motion components was confirmed on US images acquired in the vein plan (longitudinally). For veins lying in the cranial-caudal plan, the radial distribution of elastic motion was observable on transverse images and its magnitude (< 1mm) was measurable on cranial-caudal US images as a combination of anterior-posterior and cranial-caudal components. This local phenomenon was quantified on the displacement maps which showed the inhomogeneity of the motion mostly concentrated at the boundary of the sus-hepatic vein (Fig 4).

**Fig 5:**
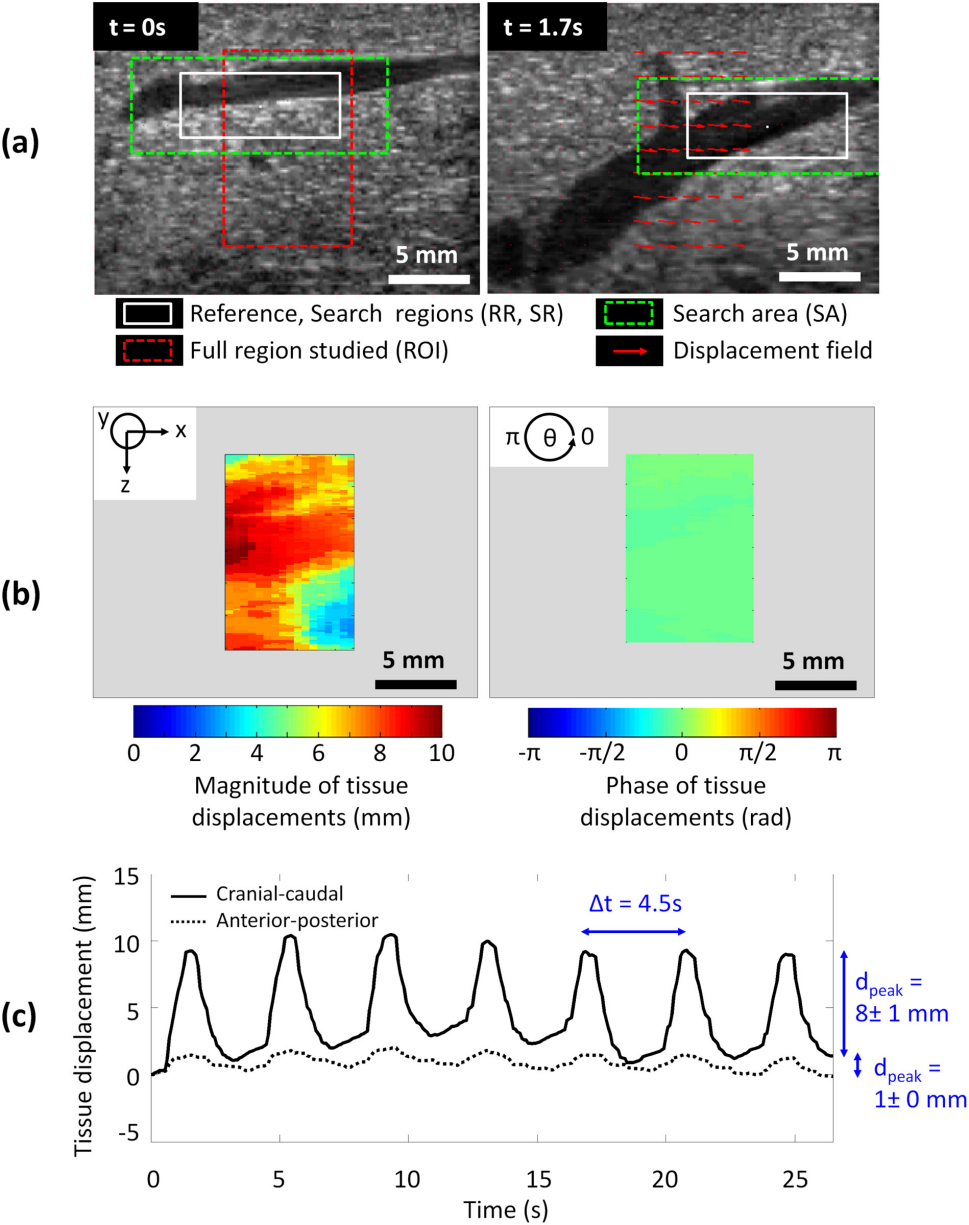
US speckle tracking of liver tissue motion surrounding a sus-hepatic vein during respiration. **(a)** 2D tracking for 2 different phases of the respiratory cycle; **(b)** 2D spatial distribution of the motion magnitude and phase: rigid motion; **(c)** 1D motion components over time.

**Fig 6:**
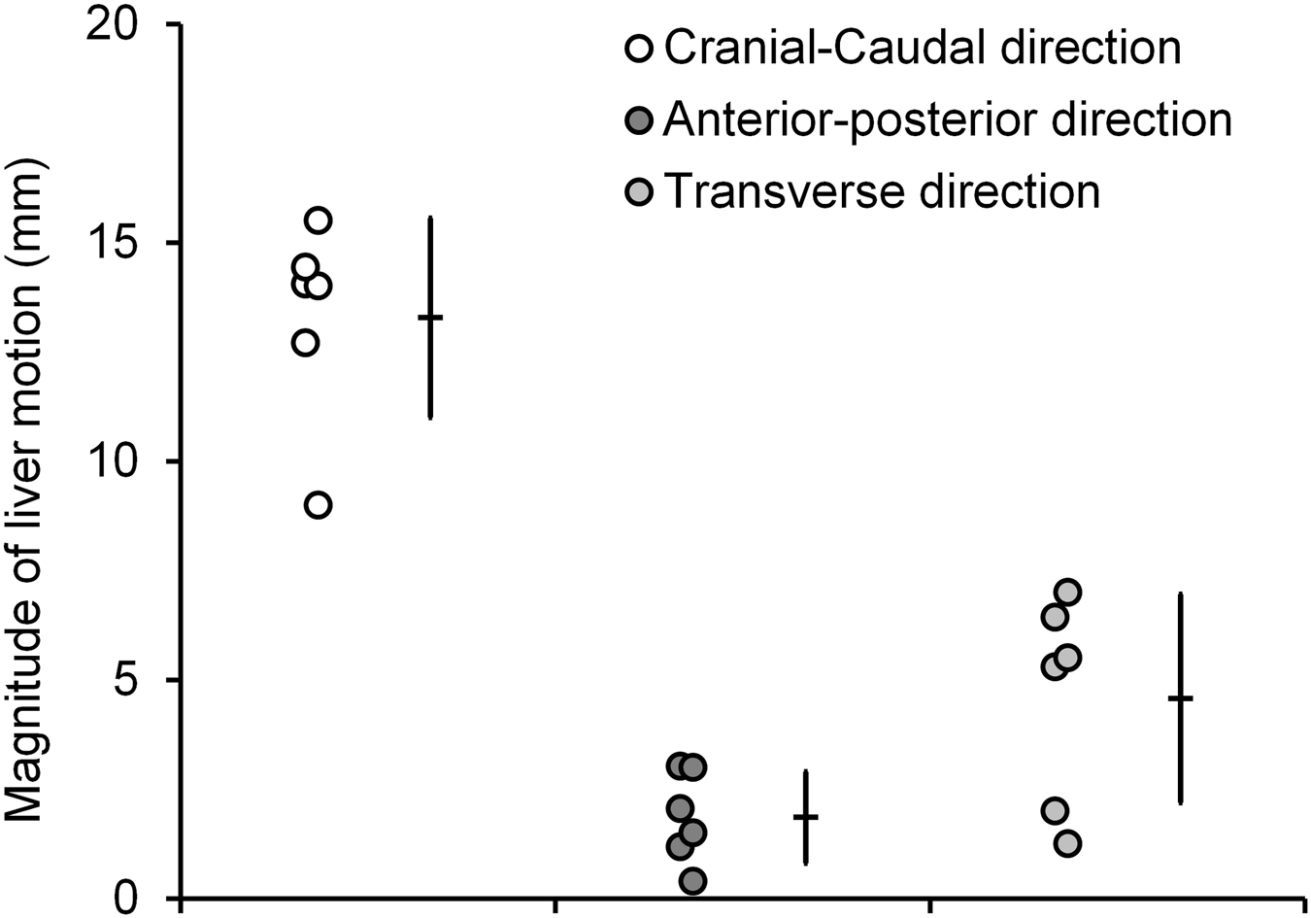
Series of *in vivo* liver motion magnitudes registered in 2 pigs on the LL, LC and RC lobes during respiration.

When using ultrasound images recorded during a period of breathing, the tracking of tissue displacement enabled direct access to the respiratory frequency (f_respiratory_ = 0.2 Hz) which was equal to the frequency imposed by the mechanical respirator (f_respirator_ = 0.2 Hz). Liver motions induced by respiratory activity were mainly encountered in the cranial-caudal direction (Ox) and spatially homogeneous at the millimeter scale in the cranial-caudal plan (xOz). As a consequence, this global displacement could be approximated to a rigid motion for further investigation of fusion modeling (Fig 5). In addition, liver motions were considered as relatively homogenous over pigs and liver lobes. Global tissue displacements tracked on cranial-caudal US images measured on average 13.3 ± 2.3 mm (range 9.0–15.5 mm) and 1.9 ± 1.0 mm (range 0.4–3.0 mm) in magnitude, respectively in cranial-caudal (Ox axis) and anterior-posterior (Oz axis) directions. The maximal instantaneous speed of liver tissue motion was 14.2 mm·s^-1^. In the transverse plane (yOz), liver movement magnitudes (Oy axis) were confirmed to be significantly lower (4.6 ± 2.4 mm (range 1.3–7.0 mm) than those measured in the cranial-caudal direction (p < 0.01) (Fig 6; Table 3).

**Table 3:** *In vivo* liver tissue motions calculated postoperatively from an incremental speckle tracking method applied on US image sequences. Data averaged over 2 pigs, with 3 measurements per pig performed on the LL, LC and RC lobes.

Liver motion direction	Motion magnitude (mm)	Mean speed (mm·s^-1^)	Maximal instantaneous speed (mm·s^-1^)	Motion tracking drift correction (mm·s^-1^)
**Cranial-caudal direction (x axis)**	13.3 ± 2.3 (range 9.0–15.5)	0.6 ± 0.1(range 0.4–0.7)	14.2 ± 2.8 (range 10.9–18.1)	0.10 ± 0.14 (range 0.00–0.30)
**Transverse direction (y axis)**	4.6 ± 2.4 (range 1.3–7.0)	_	_	_
**Anterior-posterior direction (z axis)**	1.9 ± 1.0 (range 0.4–3.0)	0.1 ± 0.0 (range 0.0–0.1)	3.0 ± 1.2 (range 0.9–4.1)	-0.02 ± 0.10 (range -0.15 –+0.06)

The magnitude of liver motion caused by the heartbeat was considered negligible (< 1 mm) when compared with liver motion caused by breathing (> 13 mm). Then, for subsequent fusion modeling in liver tissues during breathing, the total motion combining global rigid respiratory and local elastic cardiovascular motion was overall approximated to a rigid motion.

### Fusion modeling of the effects of *in vivo* liver motions on intraoperative HIFU treatments

The fusion modeling method was then implemented by combining physiological and acoustical parameters from literature with dynamic liver motion data measured *in vivo*. Different ultrasound focusing configurations have been simulated using this dynamic hybrid model in order to quantify the effect of liver motion on intraoperative HIFU therapy used as a complementary tool to surgery. All results show the effect of *in vivo* liver motions caused by respiration (average magnitude of motion in the cranial-caudal direction: 13.3 ± 2.3 mm), with motion data registered in 6 different locations (2 pigs, 3 lobes: LL, LC and RC) and calculated from ultrasound images using the incremental speckle tracking method. Sus-hepatic veins included in this study had a diameter ranging between 1 and 5 mm. All data provided describe characteristics of resulting HIFU treatments after completion of the HIFU exposures. Figures of thermal dose distributions (t_43°C_) were displayed in 2D with a minimal threshold set at 240 CEM (t_43°C_ref_). Three dimensional representations include a display of the lesion isosurface (isodose: t_43°C_ref_) and a view of the active surface of the 2 HIFU transducers used in this study.

### Modeling of liver motion effects on a “cigar-shaped” type HIFU lesion performed with spherical focusing and a 5-second single exposure

**Fig 7:**
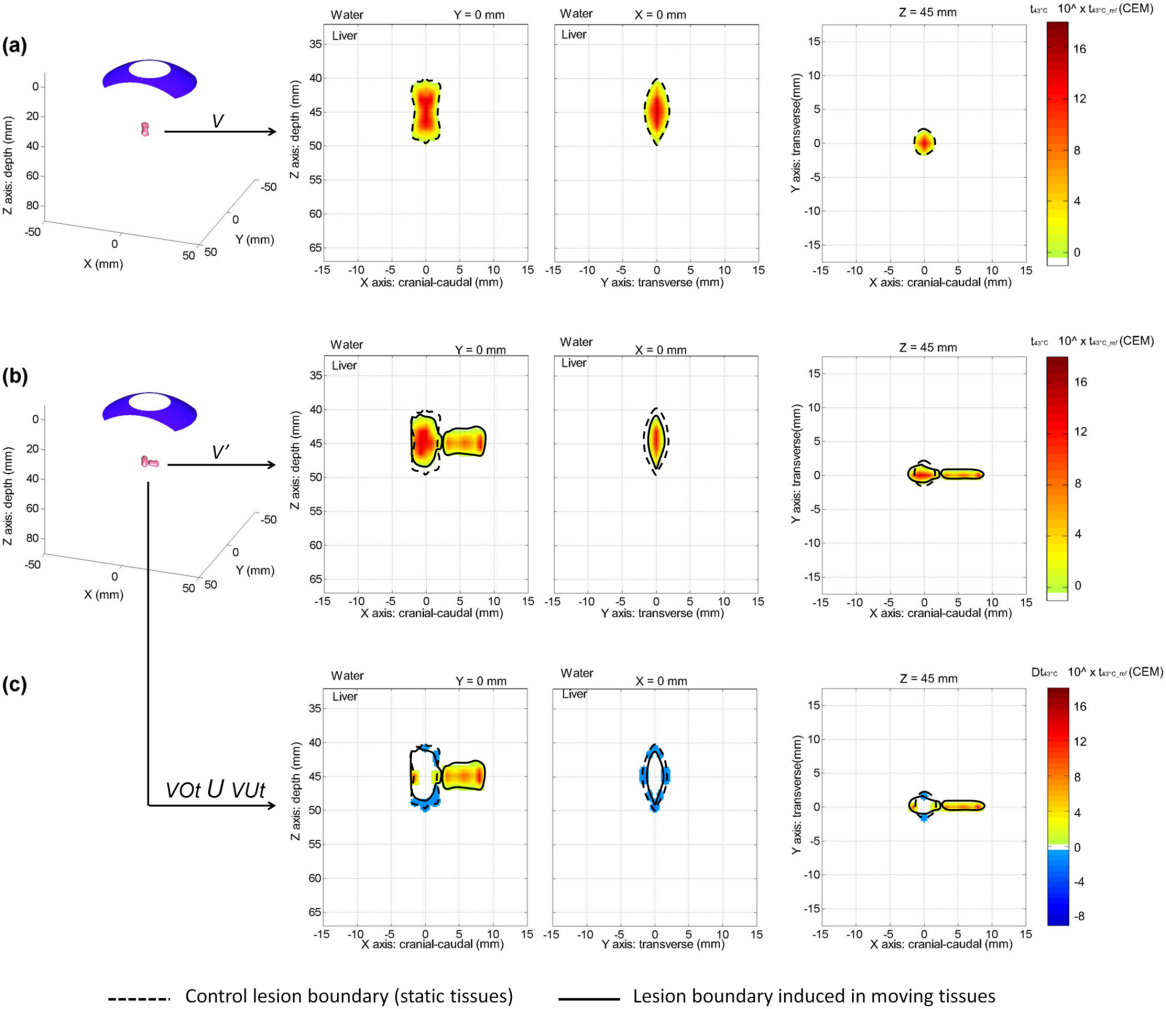
Modeling of the effects of liver respiratory motion on the creation of a millimetric single HIFU lesion. From left to right: 3D view of the equivalent time at 43°C corresponding to the HIFU lesion; 2D view of the lesion in the cranial-caudal plane; 2D view of the lesion in the transverse plane; 2D view of the lesion in the coronal plane. **(a)** Control lesion (no liver motion); **(b)** example of a single lesion generated in presence of liver motion (amplitude of 14 mm in the cranial caudal direction and of 1.5 mm in the tissue depth direction); **(c)** corresponding under- and overtreatment caused by liver respiratory motion, respectively represented by negative and positive thermal dose differences (cool and hot color scales).

In the absence of liver motion, a 5-second single ultrasound exposure generated using the spherical transducer at a power of 30W acoustic led to a single homogeneous lesion of 60 mm^3^ (Control single lesion). When including in simulations the 6 different sets of *in vivo* liver motions measured in a porcine model during respiration, the same HIFU sequence induced inhomogeneous lesions in all cases, which were spread over the tissues (Fig 7). The volume of the lesion was decreased by 23% due to respiratory motions, with an average value of 47 ± 3 mm^3^. The dramatic spatial dispersion of the lesion was not compatible with accurate treatment targeting. The major diameter of the lesion in the cranial-caudal direction extended over 13 ± 3 mm in tissues, whereas the Control single lesion without motion was confined within 4 mm. Liver tissue motion due to respiratory activity affected treatment targeting since heat deposition and temperature increase were not located at the geometric focal point of the transducer (Fig 7). The necrotized zones were off-centered in the direction of the motion by 4 ± 2 mm (range 2–7 mm) on average. Inhomogeneities were observed on heat deposition and temperature patterns which affected the lesion homogeneity, leading to the formation of a split lesion in the tissues in 83% of cases studied. The mean thermal doses, t_43°C_, in the lesion without and with motion were respectively of 1.9∙t43 CEM and 0.7∙t43 ± 0.1∙t43 CEM, corresponding to a 62% decrease within the lesion on average. When analyzing only the region of tissues initially targeted by the Control lesion, the mean t_43°C_ decreased by >80% when liver motions during respiration were accounted for. Subsequently, originally-targeted tissues were undertreated with an average VUt of 64 ± 9% (range 57–82%). Tissue motions also led to overtreatment outside the targeted region with a VOt of 40 ± 11% (range 28–59%). In potential applications requiring the generation of an isolated millimeter scale HIFU lesion, the use of gating or tracking methods to compensate thermal ablation distortions due to respiratory motion seems mandatory [1,23]. In the next section, we will see how this applies to larger HIFU treatments.

### Liver HIFU treatment during breathing: impact on centimeter scale HIFU lesions produced with spherical and toroidal focusing

**Fig 8:**
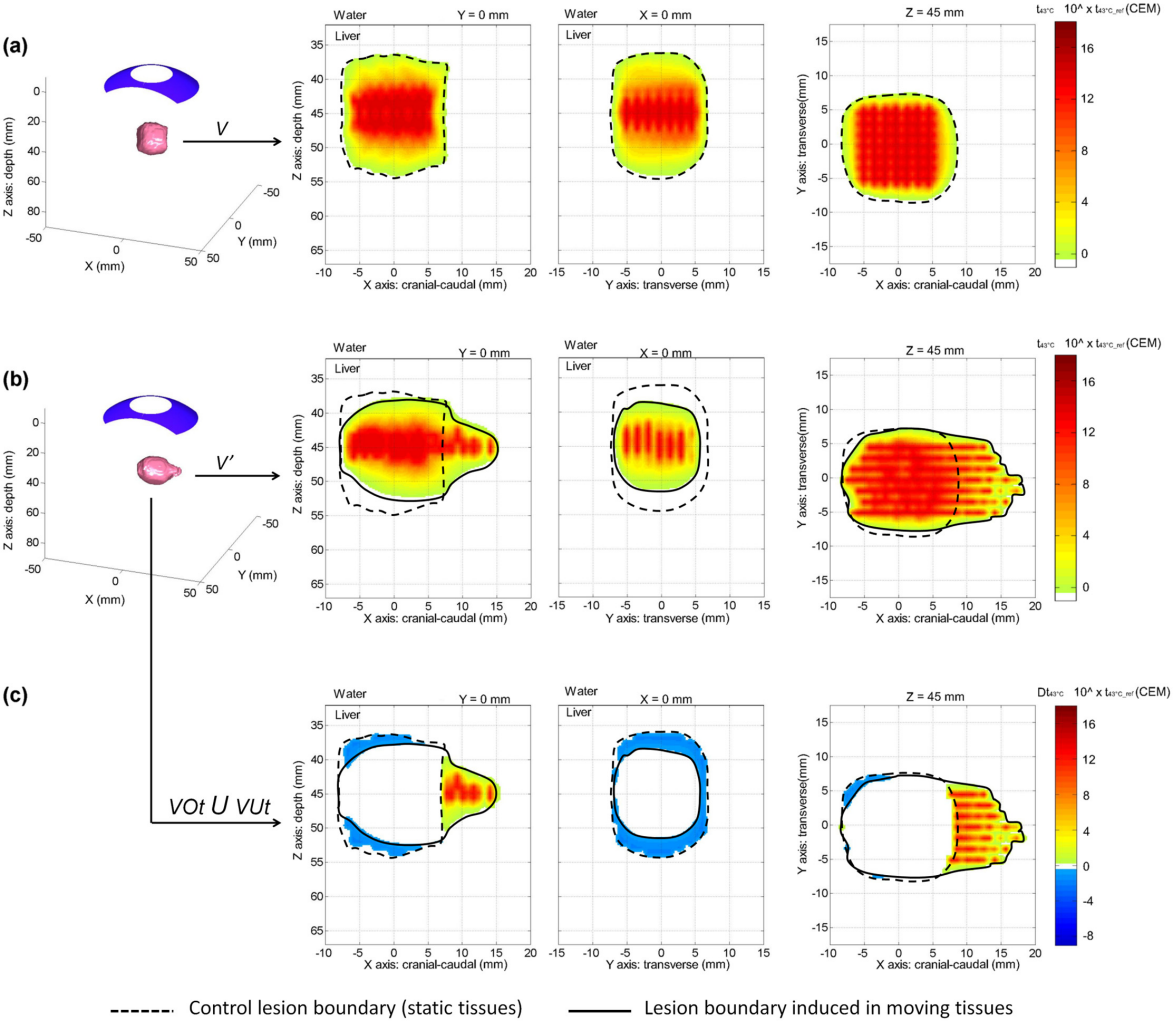
Modeling of the effects of liver respiratory motion on the creation of a large HIFU lesion made of 49 juxtaposed single lesions. From left to right: 3D view of the equivalent time at 43°C corresponding to the HIFU lesion; 2D view of the lesion in the cranial-caudal plane; 2D view of the lesion in the transverse plane; 2D view of the lesion in the coronal plane. **(a)** Control lesion (no liver motion); **(b)** example of a large lesion generated in presence of liver motion (amplitude of 14 mm in the cranial caudal direction and of 1.5 mm in the tissue depth direction); **(c)** corresponding under- and overtreatment caused by liver respiratory motion, respectively represented by negative and positive thermal dose differences (cool and hot color scales).

**Fig 9:**
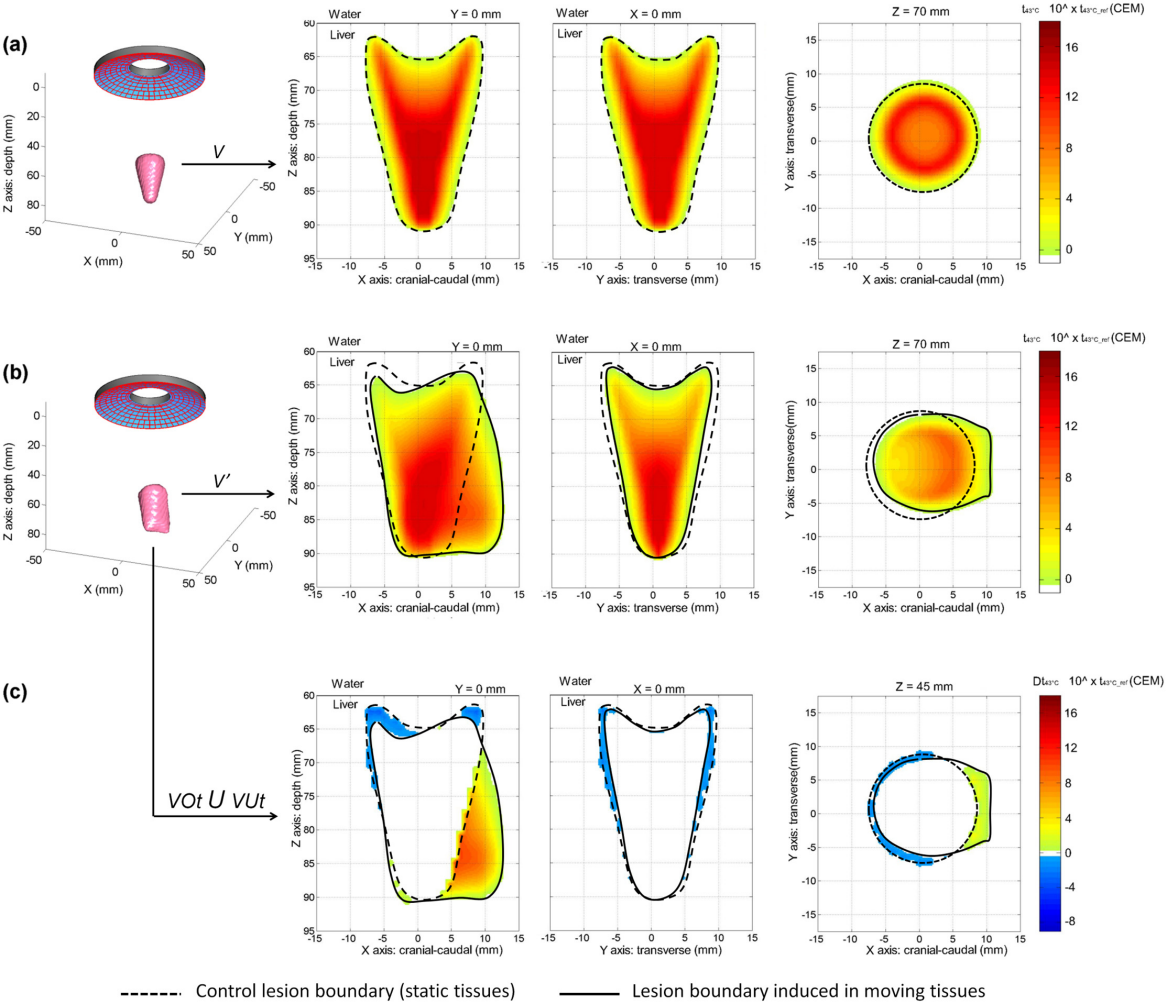
Modeling of the effects of liver respiratory motion on the creation of a large single HIFU lesion generated with a toroidal transducer. From left to right: 3D view of the equivalent time at 43°C corresponding to the HIFU lesion; 2D view of the lesion in the cranial-caudal plane; 2D view of the lesion in the transverse plane; 2D view of the lesion in the coronal plane. **(a)** Control lesion (no liver motion); **(b)** example of a single lesion generated in presence of liver motion (amplitude of 14 mm in the cranial caudal direction and of 1.5 mm in the tissue depth direction); **(c)** corresponding under- and overtreatment caused by liver respiratory motion, respectively represented by negative and positive thermal dose differences (cool and hot color scales).

**Fig 10:**
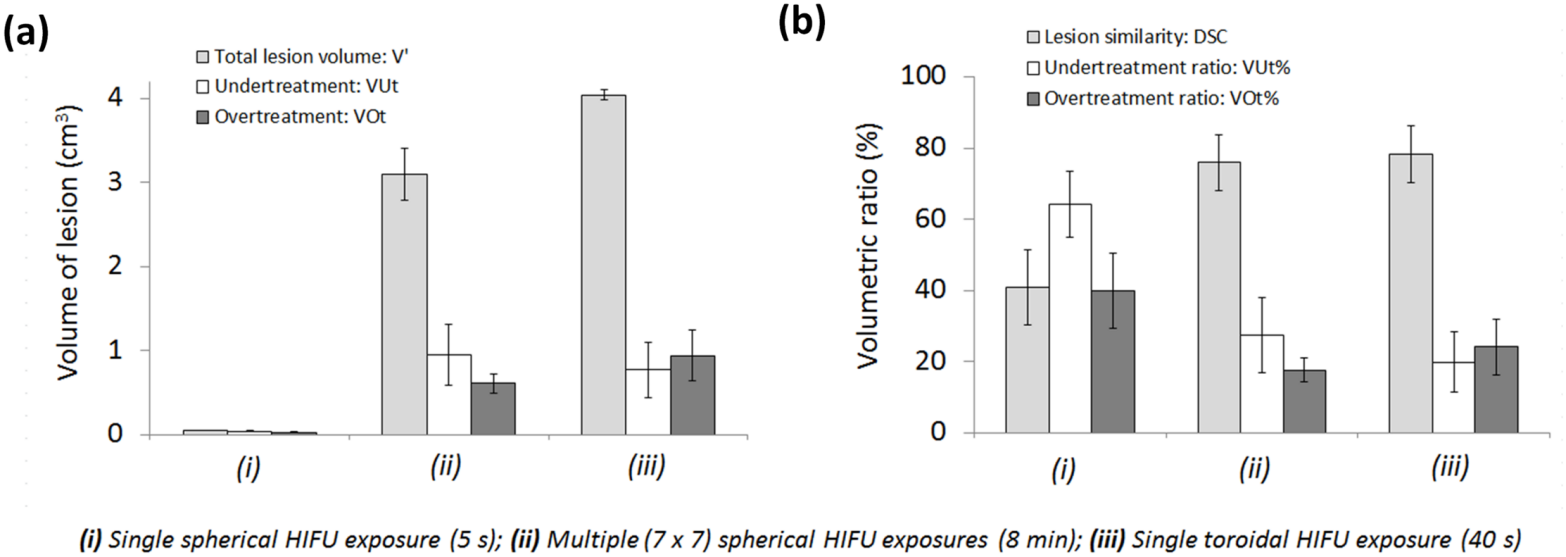
Treatment outcomes estimated by fusion modeling for 3 different ultrasound exposures sequences performed intraoperatively in liver during respiration with spherical and toroidal transducers. **(a)** Volumetric characteristics of HIFU lesions performed during respiration; **(b)** Volumetric comparison between lesions modeled with respiratory liver motion (V’) and Control lesions modeled without liver motion (V). Millimeter scale HIFU lesions (cigar-shaped type) induced by single spherical HIFU exposures were greatly affected by liver motion caused by respiration. Larger centimeter scale lesions created by juxtaposing multiple spherical HIFU exposures (7 x 7 exposures) during 8 minutes were significantly less affected as confirmed with the DSC assessment. Centimeter scale conical lesions generated with the toroidal HIFU strategy showed similar outcomes after single HIFU exposures of 40 seconds.

Here, the effects of respiratory motions on the generation of centimeter scale HIFU lesions compatible in size with thermal ablation of localized liver tumors were quantified. Without liver tissue motion, juxtaposed single HIFU lesions performed with multiple short-duration spherical HIFU exposures (49 exposures, 5s On/5s Off for each, P_ac_ = 30W) formed a large and homogeneous 3.5 cm^3^ HIFU lesion in ~8 minutes (Control lesions juxtaposed). A single toroidal HIFU exposure of longer duration (40s On, P_ac_ = 60W) was simulated in these same tissues and led to a large and homogenous 3.9 cm^3^ conical lesion (Control conical lesion). Over the 2 configurations studied, the treatment rate (volume of tissues treated per unit of time) was 14 times faster with the toroidal HIFU exposure sequence. When compared with the Control single lesion previously studied, *in vivo* liver tissue motion caused by respiration had fewer effects on the total volume of large juxtaposed and conical lesions (Figs 8 and 9). However, volumes were reshaped and stretched in the cranial-caudal direction. For spherical and toroidal HIFU exposures during respiration, average lesion volumes were respectively 3.1 ± 0.3 cm^3^ (range 2.7–3.5 cm^3^) and 4.0 ± 0.0 cm^3^ (range 4.0–4.1 cm^3^), which is a treatment rate 16 times faster for the toroidal configuration. The average DSC between lesions generated with liver motion and Control lesions (no motion) was 76 ± 8% (range 66–88%) for large juxtaposed lesions and 78 ± 8% (range 71–90%) for single conical lesions (Fig 10). With spherical HIFU exposures, the mean t_43°C_ both in the absence and presence of liver motion were 5.9∙t43 CEM and 2.4∙t43 ± 0.5∙t43 CEM respectively, which is an average t_43°C_ decrease of 72%, comparable to the decrease previously observed in the case of a millimeter scale single lesion. With toroidal HIFU exposures, the mean t_43°C_ in the Control conical lesion was 17.8∙t43 CEM and decreased by 78% (4.0∙t43 ± 2.9∙t43 CEM) with respiratory motions. In most of the tissues originally targeted by HIFU, however, t_43°C_ was maintained over the minimum threshold t_43°C_ref_, enabling generation of irreversible damage. Large HIFU lesions were created in the liver and stretched in the cranial-caudal direction while remaining homogeneous. Without motion correction, 28 ± 11% (range 11–41%) of the targeted tissues were undertreated with the spherical configuration. Using the toroidal HIFU device enabled slightly decreasing the average VUt% caused by liver tissue motions down to 20 ± 8% (range 7–29%) of the targeted tissues. At the same time, liver motions modified the energy distribution outside the region originally targeted, resulting in overtreatments. Overtreated tissues extended in the direction of the liver motion (cranial-caudal) and over a distance comparable to the amplitude of tissue displacements. For spherical and toroidal HIFU exposures, the average VOt% was respectively 18 ± 3% (range 12–21%) and 24 ± 8% (range 12–31%) (Fig 10). The necrotized zones were off-center in the cranial-caudal direction by 4 ± 1 mm (range 2–5 mm) and by 3 ± 1 mm (range 1–5 mm) for spherical and toroidal configurations respectively. While reducing treatment time significantly, the treatment efficiency and accuracy achieved with a single toroidal HIFU exposure were comparable to those simulated for a juxtaposition of multiple spherical HIFU exposures with and without respiratory motion. All parameters on simulated HIFU lesions are summarized in Table 4.

**Table 4:** Liver motion effects on HIFU treatment key parameters. Lesions performed with spherical and toroidal HIFU exposure sequences in liver.

	Ellipsoidal single lesion (Spherical transducer)		Large treatment: 49 ellipsoidal single lesions (Spherical transducer)		Large treatment: Conical single lesion (Toroidal transducer)	
Nature of liver motions accounted in simulations	No motion	Respiratory motion	No motion	Respiratory motion	No motion	Respiratory motion
**Lesion volume (cm** ^**3**^ **)**	0.06	0.05 ± 0.00(- 24%)	3.4	3.1 ± 0.3(- 10%)	3.9	4.0 ± 0.0(+ 4%)
**Treatment rate (cm** ^**3**^ **·min** ^**-1**^ **)**	0.74	0.56 ± 0.03 (- 24%)	0.42	0.38 ± 0.04 (- 10%)	5.80	6.06 ± 0.09 (+ 4%)
**Lesion similarity: DSC (%)**	_	41 ± 11	_	76 ± 8	_	78 ± 8
**Undertreatment: VUt% (%)**	_	64 ± 9	_	28 ± 11	_	20 ± 8
**Overtreatment: VOt% (%)**	_	40 ± 11	_	18 ± 3	_	24 ± 8
**Off-centering (mm)**		4 ± 2		4 ± 1		3 ± 1
**Lesion homogeneity**	Yes	No: 83% of the cases	Yes	Yes: all cases	Yes	Yes: all cases
**Mean t**_**43°C**_ **inside the lesion (CEM)**	1.9∙t43	0.7∙t43 ± 0.1∙t43 (- 62%)	5.9∙t43	2.4∙t43 ± 0.5∙t43 (- 72%)	17.8∙t43	4.0∙t43 ± 2.9∙t43 (- 78%)
**Cranial-caudal main diameter (mm)**	4	13 ± 3 (+250%)	15	23 ± 3 (+ 51%)	16	15 ± 1 (- 8%)
**Cranial-caudal minor diameter (mm)**	2	3 ± 0 (+38%)	15	11 ± 2 (- 25%)	7	15 ± 3 (+ 116%)
**Transverse main diameter (mm)**	3	2 ± 0 (- 53%)	14	13 ± 0 (- 8%)	16	14 ± 1 (- 14%)
**Transverse minor diameter (mm)**	2	1 ± 0 (- 80%)	12	9 ± 1 (- 29%)	7	5 ± 1 (- 28%)
**Main depth (mm)**	7	6 ± 0 (- 18%)	18	15 ± 2 (- 21%)	25	24 ± 1 (- 4%)

### Fusion modeling of *in vivo* HIFU experiment

**Fig 11:**
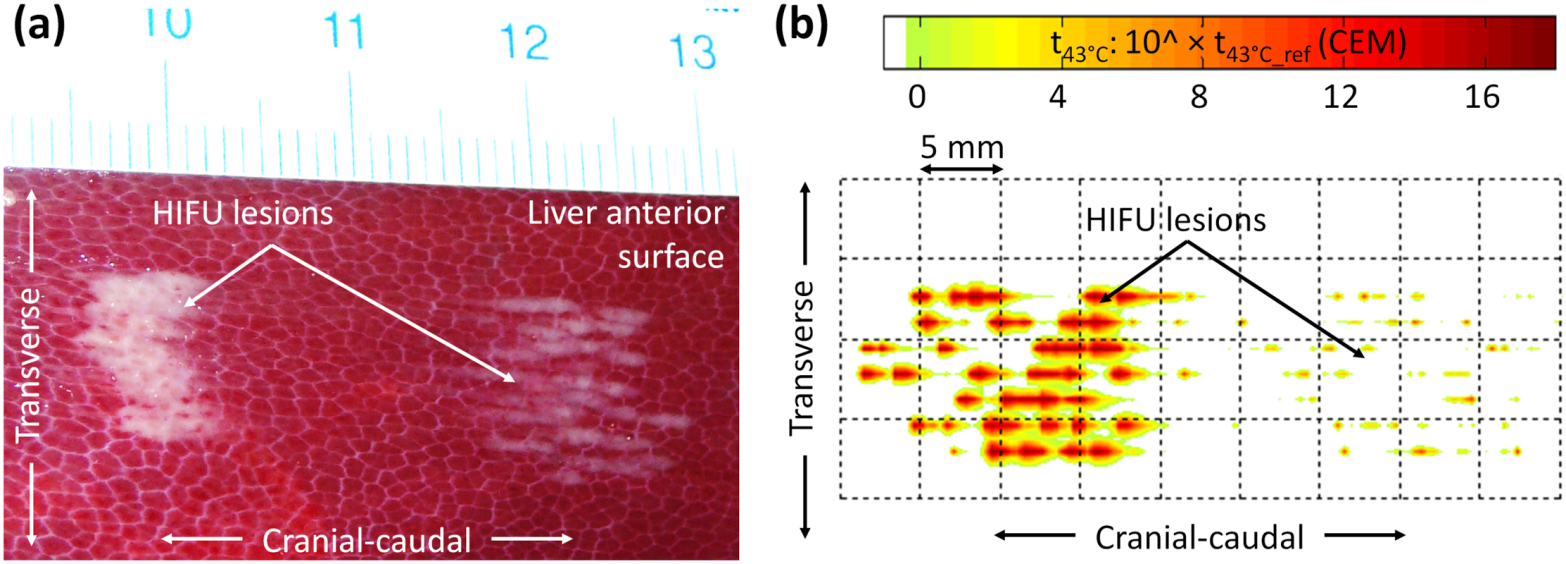
Fusion modeling of HIFU treatment versus experimental *in vivo* HIFU in a porcine model. Post-treatment fusion modeling merged real *in vivo* liver motion acquired intraoperatively during HIFU treatments (US speckle tracking) and numerical data from the BHTE solving program. Example with the spherical HIFU focusing strategy studied: *in vivo* HIFU ablations were generated with a juxtaposition of 7x7 lesions during breathing (total exposure time: 8 minutes, P_ac_ = 30 W). The HIFU lesion was stretched along the motion direction (cranial-caudal) and individual cigar-shaped type lesions were not juxtaposed homogenously. View of the anterior surface of the liver **(a)** *in vivo* result (Day 0); **(b)** fusion modeling result.

**Fig 12:**
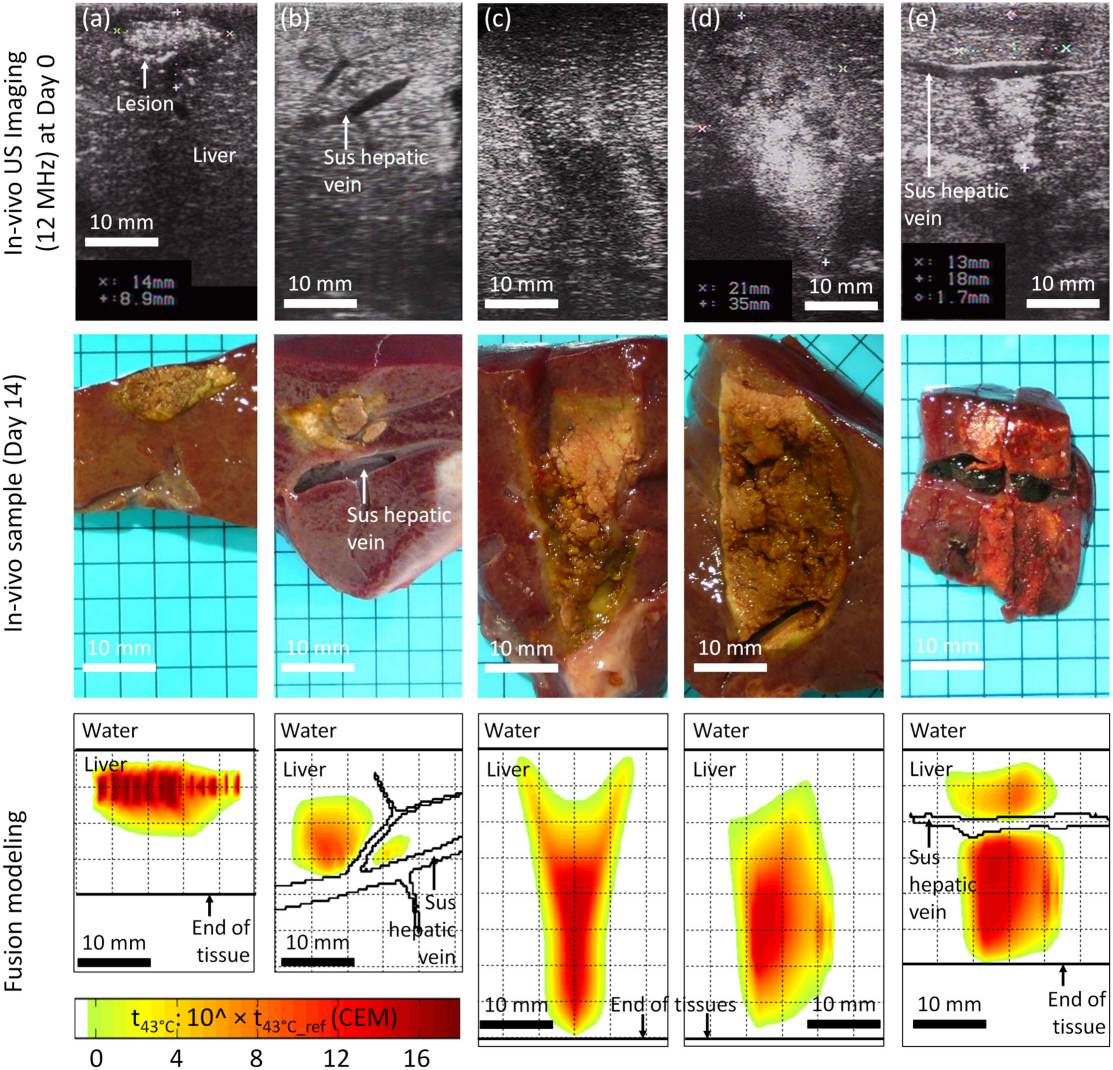
Preliminary *in vivo* validation of fusion modeling in a porcine model for 2 ultrasound focusing strategies: toroidal and spherical HIFU. Post-treatment fusion modeling merged real *in vivo* liver motions acquired intraoperatively during HIFU treatments and realistic geometries of sus hepatic veins. Macroscopic analyses were performed on liver tissue samples removed 14 days after HIFU sessions. **(a to b)** Spherical focusing: 2 examples of *in vivo* HIFU ablations generated with a juxtaposition of 7x7 lesions during breathing (total exposure time: 8 minutes, P_ac_ = 30 W); **(a)** in moving homogeneous liver tissues; **(b)** in moving liver tissues in presence of a sus-hepatic vein. **(c to e)** Toroidal focusing: 3 examples of *in vivo* HIFU ablations generated with one single exposure during breathing (total exposure time: 40 seconds, P_ac_ = 50 W); **(c)** in stationary homogeneous liver tissues (40-second apnea); **(d)** in moving homogeneous liver tissues; **(e)** in moving liver tissues in presence of a sus-hepatic vein.

In this last series of investigations, HIFU exposures were performed *in vivo* in a porcine model with the spherical and toroidal HIFU focusing strategies studied in simulation. Postoperative fusion modeling was performed successfully by integrating *in vivo* liver motion observed during the HIFU experiments, allowing direct comparison between fusion modeling outputs and experimental results for each HIFU treatment. Overall, the fusion modeling of HIFU treatments showed very similar results to those observed macroscopically on real tissues during *in vivo* experiments, for both spherical and toroidal HIFU strategies with and without respiratory motions. The size and shape of the simulated and experimental HIFU lesions were particularly in agreement when the liver regions targeted were superficial, as observed at Day 0 just after the end of the HIFU exposures at the surface of the organ (Fig 11). HIFU lesions estimated postoperatively with fusion modeling were also comparable to *in vivo* thermal lesions observed at Day 14 after liver dissections in deep tissues (range: 0 to 40 mm) (Fig 12). A part of the overestimation made with the simulations may be due to the fact that the moving liver surface was not perfectly flat and horizontal in the pig abdomen *in vivo*, which could lead to a HIFU focal region being periodically ahead of the tissue. Macroscopic examinations performed 14 days after the HIFU treatments allowed validating the accuracy of the fusion modeling and its ability to estimate, despite interfering motion, the actual conformations of *in vivo* HIFU lesions once they were completely established within the tissues. Experimental analyses confirmed the deleterious effects of respiratory liver motions which prevent accurate reconstruction of large HIFU lesions when using juxtapositions of multiple millimeter scale “cigar-shaped” type lesions. HIFU ablations generated during respiration after 8 minutes and 49 lesions were stretched along the cranial-caudal direction and their homogeneity could be challenged by large motion amplitudes (> 10 mm) or by the presence of vascular structures (Fig 12a and 12b). According to *in vivo* observations, the main effects of liver motions on spherical HIFU treatments could be recreated by the fusion modeling technique for different treatment conditions: targeting of various tissue depths ranging from 0 to 15mm, HIFU exposures in moving tissues (respiration), homogenous and heterogeneous (absence or presence of large sus hepatic veins, diameter ranging between 1–5 mm). The fusion modeling was also successful in predicting thermal lesions performed with the toroidal HIFU system, which provided preliminary *in vivo* validation of the presented technique for 2 different HIFU focusing strategies (Fig 12c–12e). Macroscopic analyses confirmed the potential of the toroidal focusing for fast generation (40s) of large HIFU lesions during intraoperative procedures, in presence of liver tissue vascularization (vessel < 5mm in diameter) and motion (amplitude < 15 mm). Lesion main diameters (15–20 mm) and lesion depths (20–35 mm) varied with the lobe thickness and the transducer-to-tissue distance.

## Discussion

In this paper, an US image-based dynamic fusion modeling method for predicting HIFU treatment performances in realistic *in vivo* conditions has been presented and preliminarily tested *in vivo*. This method was used to study the quantitative effects of *in vivo* liver motion on intraoperative HIFU treatments in a porcine model. The intraoperative open procedure allowed high resolution ultrasound imaging of the liver in movement and enabled simulating the dispersion of the heat deposition due to respiratory motion effects. The main liver motion due to respiration in the porcine model occurred in the cranial-caudal direction as observed in human [62], confirming the interest of this animal model for studying the management of liver motions at a preclinical level. Proper estimations of the motion components were achieved with standard 2D ultrasound B-mode imaging, which could facilitate the transfer of this technique on most of the commercialized ultrasound imaging systems and support its integration on USgHIFU medical devices. US speckle tracking of *in vivo* liver motion allowed detection of motion components showing very different characteristics throughout the liver parenchyma. The main motion of a liver lobe due to respiration (several millimeters in magnitude) was homogeneous enough throughout tissues to be seen as a rigid periodic global motion in our application, while sub-millimeter elastic displacements due to cardiovascular activity co-existed locally close to sus-hepatic veins. The accurate detection of respiratory liver motions on standard 2D B-mode US images is of particular importance for developing fusion modeling applications dealing with the predictions of *in vivo* HIFU performances during respiration. In the presented paper, method of cross correlation between 2D ultrasound images based on speckle tracking enabled access to relevant values of respiratory frequency (0.2 Hz). Liver motion magnitudes due to respiration (9–16 mm) were comparable to data reported in the porcine model, obtained with electromagnetic tracking methods detecting the movement of sensors attached to the surface of the liver (Frequency: 0.23 Hz, magnitude: 5–6 mm) [63]. These data were also comparable to those measured by scintigraphy in human (Weiss et al. (1972) [6], magnitude: 16–17 mm; Harautz et al. (1979) [7], magnitude: 13 mm (min. 9—max. 28 mm), frequency: 0.33 Hz (min. 0.14—max. 0.50 Hz)), by tomography (CT) scan (Beddar et al. (2007) [8], magnitude: 7.5–17.5 mm), or by MRI (Kirilova et al. (2008) [9], magnitude: 15.5 mm (min. 6.9—max. 35.4 mm)).

One advantage of studying fusion modeling, in the context of intraoperative HIFU interventions assisting standard surgery, is the possibility to use high resolution ultrasound imaging modalities during open surgery. Sub-millimeter deformations of sus-hepatic veins occurring during dilatation/compression cycles were detectable and relevant values of heart beats frequency (0.96 Hz) were measured which were consistent with standard ECG monitoring [64]. This parameter was not accessible in the study reported by Olbrich et al. (2005) [63] since liver motions magnitude due to cardiovascular activity was lower than noise measurement. These data were also not mentioned in the human studies previously cited. In the presented study, sub-millimeter motion due to the heartbeat is negligible compared to respiratory motion in the liver and was considered as less critical for the establishment of large HIFU ablations. Fusion modeling could however benefit from those data for other HIFU applications involving finer ablations or more complex treatment plans, as previously proposed with US-guided transoesophageal cardiac HIFU ablation [57], provided that current works on the miniaturization and integration of US transducers allow better access to high resolution 3D US imaging. Although proper acquisitions could be made of the respiratory and cardiovascular components involved in real liver motion, the use of standard 2D images was a limitation in this study as it required identifying one main plan covering most of the organ motions. The fusion modeling method could then benefit from the emergence of dual-mode ultrasound and the development of 3D US imaging for accounting more complex tissue motions. Previous investigations have indeed confirmed that US speckle tracking could be used successfully on 2D multi-planar ultrasound images for detecting accurately 3D motions [10]. This technique has shown promise for the description of 3D displacements. The ability to track sub-millimeter displacements, as can be done when performing ultrafast shear waves elastography, may also offer various possibilities of investigations for studying other aspects of the fusion modeling such as, for instance, the use of realistic *in vivo* tissue elasticity in simulations.

In the presented work, the fusion modeling was used to study the performance of intraoperative HIFU treatments during respiration, and more particularly, the interest of using toroidal HIFU focusing rather than classical spherical HIFU strategies for liver ablation. Our team has previously studied HIFU treatments using a toroidal-shaped transducer, and has shown that it could represent a promising alternative for treating colorectal liver metastases [41]. The principal interest lies in the possibility of treating hepatic parenchyma in a short period of time (ablation of 5–7 cm^3^ in 40 s) without any organ puncture or blood contact. The intraoperative approach selected for the toroidal HIFU treatment (after surgical laparotomy) makes it possible to reach all regions of the liver without penetrating the hepatic capsule, particularly in Human for whom all liver sectors are accessible by this strategy [65]. This configuration also enables the protection of surrounding organs and eliminates the risk of secondary lesions. In addition, as suggested for radiofrequency and cryosurgery ablation procedures, combining hepatic resection with HIFU ablation could expand the number of patients who may be candidates for liver-directed surgical therapy [66,67]. The use of an extracorporeal HIFU device is clinically feasible for the treatment of hepatocellular carcinoma, but accessing all regions of the liver from outside the rib cage remains challenging and the use of partial rib resection to create a better acoustic window was showed to favor complete tumor HIFU ablations [35,68,69]. Beside respiratory movements, inhomogeneous attenuation and phase aberration in the rib-cage can create severe ultrasound energy dispersion in the focal region [3] or produce secondary lesions in surrounding tissues (such as skin burns or gastric lesions).

Despite the ideal conditions of ultrasound transmission provided during intraoperative open procedures, the fusion modeling results highlighted significant deleterious effects of respiratory motions on the homogeneity of millimeter scale thermal lesions induced with single, short-duration spherical HIFU exposures. Although juxtapositions of multiple single lesions allowed significant reduction of this effect, the accumulation of thermal energy in the tissues using this HIFU exposure strategy was time-costing and required bulky instrumentations to move the transducer according to the treatment plan. In addition, the targeting accuracy remained slightly affected, according to the treated zone which was distorted in the cranial-caudal direction and off-center of the targeted region by several millimeters. Nevertheless the reshaped lesions showed reasonable similarity (75%) to the Control lesion without motion. Results obtained in fusion modeling with the toroidal HIFU device show that the elementary conical-shaped lesion created in one shot was slightly affected by tissue motion (78% of similarity with the Control lesion), while the major advantage of this strategy was to significantly increase the treatment rate (ratio > 10). The volume of tissue exposed to a lethal t_43°C_ remained homogeneous, despite the widening of the lesion at its extremity, and undertreatments were slightly lower than those observed when juxtaposing multiple spherical HIFU exposures.

Validation of this dynamic model will be effective if the consistency of simulation results with *in vivo* observations can be demonstrated for various experimental configurations. In the present paper, initial investigations have been introduced and provided a preliminary evaluation of the method for 2 specific spherical and toroidal HIFU strategies. Overall observations indicate that fusion modeling predictions of intraoperative *in vivo* HIFU treatments performed during respiration were in-line with observations made experimentally. These analyses were reinforced by confirming the alignment between modeling and *in vivo* trials for radically different HIFU focusing strategies and various tissue environments (homogeneous, vascularized). Thermal lesions induced during respiration were confirmed *in vivo* to be distorted mainly in the cranial-caudal direction, which supports our first assumption that considering a global 2D rigid respiratory motion is sufficient to account for most liver motion effect on intraoperative HIFU treatment. This also suggested that real-time speckle tracking method implemented in 2D during intraoperative USgHIFU exposures in liver would be sufficient to compensate for most effects induced by respiratory motion and to prevent distortion of the HIFU lesion (mechanical compensation for motion, HIFU beam steering using dynamic focusing, motion gating with HIFU exposures). The accuracy of fusion modeling might be currently challenged by several parameters, and further *in vivo* validations are undoubtedly necessary to confirm the robustness of the method for predicting liver motion effects on HIFU treatments in realistic *in vivo* conditions. Firstly, the *in vivo* determination of a precise thermal dose threshold for the appearance of irreversible thermal damage in biological tissues remains challenging, and a range of values are currently available in the literature [58], which vary according to the organ studied, but can also vary for a given organ with the HIFU exposure and measurement conditions. Secondly, the model of equivalent time at 43°C used to quantify thermal damage induced at high temperatures by HIFU (T>60°C) is derived empirically from isoeffects observed with low temperature hyperthermia (42°C<T<60°C). Thirdly, the interaction between HIFU and biological tissues can generate both thermal and mechanical effects. The fusion modeling method presented in this paper estimates the damage caused by thermal effects only, and mechanical damages which could for instance arise from boiling cavitation, are not considered. By including *in vivo* tissue motions in modeling with certain assumptions (ex: respiratory motion approximated to a rigid motion in the cranial-caudal direction), the uncertainty of achieving irreversible thermal damage similarly in simulation and during experiments increases, particularly in zones of tissues where t_43°C_ is close to the minimum thermal dose threshold (240 CEM). Disparity in energy distribution is also increased by the intermittent nature of the HIFU sequence used with the spherical transducer. Predictions of lesion volumes could potentially be affected by this uncertainty, and the volume of necrosis obtained *in vivo* might be over-/underestimated by the modeling. The tissue motion phase at which HIFU exposures began may also have an impact on predictions. Although this effect was considered as minor due to the long treatment durations used for inducing centimeter scale lesions in this study (>8 periods of the main motion), accounting for this parameter could reduce some discrepancies seen between experiments and simulation. Secondly, regarding treatment accuracy, targeting quality might be affected drastically if the intense part of necrosis is shifted significantly in the cranial-caudal direction. Another source of prediction error can arise in the calculation of the acoustic pressure field in the presence of heterogeneous moving tissues. In the presented study, these effects have been considered to be negligible when introducing single vessels in the model (<5mm in diameter), since they were mostly oriented perpendicularly to the ultrasound propagation direction. To maintain reliable predictions of *in vivo* HIFU lesion formation in more complex heterogeneous structures such as in highly vascularized tissues (ex: cavo-hepatic junction in the liver) or large blood cavities (ex: atria and ventricles in the heart), recalculating the acoustic pressure field for each phase of tissue motion may be critical. Although the proposed method made an accurate estimation of HIFU lesion creation in perfused tissues and in the presence of a large blood vessel (> 1 mm and < 5 mm in diameter), the influence of perfusion variations according to temperature was neglected and might be better accounted for if predictions require refinement. Finally in the present work, fusion modeling does not integrate any parameter accounting for biological evolutions of HIFU lesions such as apoptosis, which can occur at the boundary of the lesion in the transition zone between necrotized and native tissues.

Preliminary *in vivo* validations of fusion modeling results demonstrate an advantage of continuous toroidal HIFU exposures over intermittent spherical HIFU strategies, in developing intraoperative liver treatments during surgery. The ability to generate a large ablation during respiration with a fast single HIFU exposure in the sub-minute range (40s) could enable use of the device by hand [40], without the need for an additional system of displacement for mechanical tracking and without using apnea. This represents an advantage over conventional highly focused HIFU strategies, for which real-time motion tracking or gating methods are usually needed for compensating deleterious effects of tissue motions [1,2,3,4]. Difficulties associated with motion have not only been observed in the area of HIFU treatments. Other techniques have been set to compensate or attenuate movements. For instance, Active Breathing Control (ABC) methods involving apnea of 5–10 seconds to suppress motion caused by respiration have been proposed during radiotherapy for cancer treatments in lung, pancreatic or hepatic metastasis [70,71,72]. This solution has been preliminarily used by our team during previous preclinical studies with the toroidal HIFU transducer [73]. However, the duration of apnea periods reported in these studies was longer as mechanical ventilation was interrupted for at least 40 seconds to obtain an elementary conical HIFU lesion. Although this method was well tolerated by animals and ensured accurate treatment targeting, this approach may show limitations on a clinical level. In our study focusing on liver motion effects on HIFU treatments, we observed reasonable similarities between toroidal lesions with and without movements, both with fusion modeling and during *in vivo* experimentation. To use fusion modeling as a robust quantitative method *in vivo* in liver or in other organs will, however, require further investigation, especially exploring methods to improve access to *in vivo* tissue parameters in real-time and methods to analyze the HIFU lesion evolution over time in tissues.

Based on these results and previous preclinical investigations, a proof-of-concept clinical study is ongoing for the treatment of liver metastases and is assessing the performances of the toroidal HIFU device used by hand without mechanical tracking nor apnea. The intraoperative approach during surgery, and the anatomy of the human liver together enable direct access to the organ and careful isolation of the liver by placing surgical pads in the patient’s abdomen. Clinically, this was found to be a sufficient alternative to removing apnea from the procedure while ensuring accurate treatment during breathing.

To conclude, a dynamic US image-based fusion modeling method to estimate the effects of real *in vivo* liver motion on HIFU treatments was presented. The technique combines numerical data with reliable dynamic liver motion data obtained from ultrasound speckle tracking. Global rigid liver motions and local elastic tissue deformations are accessible and can be used for modeling HIFU lesion formation in *in vivo* biological tissues. By accounting for real *in vivo* tissue motions, the fusion modeling provides new data and realistic estimations of HIFU ablations in accordance with *in vivo* observations. Currently, this method can be useful post-operatively, to anticipate, compare and improve HIFU performances (focusing strategies, exposure sequences and treatment planning) by considering realistic tissue environment. The toroidal HIFU strategy, for instance, has showed some advantages for treating liver tissues intraoperatively during respiration. The treatment is faster than a strategy involving multiple juxtaposed millimeter scale lesions, similarly affected by organ motion and can be targeted manually, which shows promise for the development of HIFU treatment applications during an open procedure. With the emergence of 3D image fusion and Augmented Reality for guiding surgical interventions, and current ongoing works to develop robust US-based HIFU monitoring techniques (US thermometry, elastography), more advanced versions of US image-based dynamic fusion modeling strategies could also be interesting in the future for assisting USgHIFU thermal ablations.
